# A Framework for Global Collaborative Data Management for Malaria Research

**DOI:** 10.4269/ajtmh.15-0003

**Published:** 2015-09-02

**Authors:** Juan B. Gutierrez, Omar S. Harb, Jie Zheng, Daniel J. Tisch, Edwin D. Charlebois, Christian J. Stoeckert, Steven A. Sullivan

**Affiliations:** Institute of Bioinformatics and Department of Mathematics, University of Georgia, Athens, Georgia; Department of Biology, University of Pennsylvania, Philadelphia, Pennsylvania; Institute for Biomedical Informatics, University of Pennsylvania Perelman School of Medicine, Philadelphia, Pennsylvania; Department of Genetics, University of Pennsylvania Perelman School of Medicine, Philadelphia, Pennsylvania; The Center for Global Health and Diseases, Case Western Reserve University School of Medicine, Cleveland, Ohio; Department of Medicine, University of California, San Francisco, California; New York University Center for Genomics and Systems Biology, New York, New York

## Abstract

Data generated during the course of research activities carried out by the International Centers of Excellence for Malaria Research (ICEMR) is heterogeneous, large, and multi-scaled. The complexity of federated and global data operations and the diverse uses planned for the data pose tremendous challenges and opportunities for collaborative research. In this article, we present the foundational principles for data management across the ICEMR Program, the logistics associated with multiple aspects of the data life cycle, and describe a pilot centralized web information system created in PlasmoDB to query a subset of this data. The paradigm proposed as a solution for the data operations in the ICEMR Program is widely applicable to large, multifaceted research projects, and could be reproduced in other contexts that require sophisticated data management.

## Introduction

**Figure 1.**
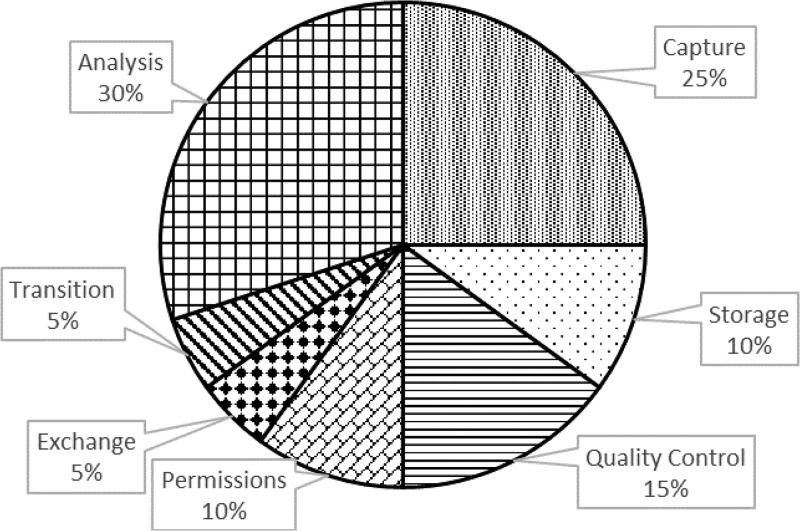
Data management for a complex project such as the International Centers of Excellence for Malaria Research (ICEMR) Program should consider all phases in the life cycle of data, even after the project has ended. This graph illustrates the comparative amount of energy one might allocate to each phase, and is based on estimates from a single ICEMR (non-Amazonian Latin America). Specifically, “capture” refers to information collection, “storage” refers to the physical and digital repositories that contains the information, “quality control” refers to the procedures in place that guarantee integrity of the data, “permissions” refer to mechanisms in place to restrict access to information, “analysis” refers to pipelines for quantitative analysis of the data, “exchange” refers to processes that allow data to be shared, and “transition” refers to processes in place to ensure that data and analysis pipelines are available after the research projects have finished.

Success in life sciences research depends on the ability to properly manage, integrate, and interpret complex data sets.^1^ The International Centers of Excellence for Malaria Research (ICEMR) Program is a global network of 10 independent research Centers in malaria-endemic settings in 19 countries spanning Latin America,^2^ Africa,^3^ Asia^4^ and the Pacific.^1^ As each center is meant to have a regional focus, some conduct research in more than one country. In addition to research projects carried out by individual centers, opportunities for collaboration across the network exist and are encouraged. The ICEMR Program aims to understand the epidemiology, pathogenesis, and transmission patterns of malaria in different geographic regions. Data generated by the ICEMR Program during the course of the research activities are heterogeneous and multi-scaled.^2^ For example, ICEMRs are employing an array of study designs including active, passive and reactive case detection, matched case–control, longitudinal and control intervention studies, entomological surveys, spatial analyses, as well as laboratory studies employing a variety of molecular markers. The ICEMR data management setting consists of a federation of independent information systems, central repositories, and procedures to enforce best practices with regards to data capture, analysis, exchange, and persistence (Figure 1

). Such complex data management operations require consideration of^1^ standards for collecting and managing data,^2^ ethical issues of consent and privacy,^3^ quality assurance (QA) and quality control (QC) of data,^4^ software quality attributes^5^ security and authentication,^6^ research operations and analysis needs, and^7^ transition to a post-project state of data access and preservation.^3^,^4^

Stewardship of data is intended not only to support concomitant research activities, but also to support the development of future analyses. To ensure wider dissemination of ICEMR data, close interactions have been established with the relevant National Institutes of Health (NIH)–funded bioinformatics resources including VectorBase (the functional genomic resource for vectors of eukaryotic pathogens, including *Plasmodium*) and PlasmoDB (the functional genomic resource for *Plasmodium* parasites).^5^,^6^ Coherent data capture, organization, processing, and preservation will ensure that the ICEMRs are productive beyond the context of initial collection and analysis, thanks to a controlled flow of data channeled through appropriate procedures and devices.^7^ This article highlights the collaborative process that developed systems and procedures to 1) perform this global research program, 2) manage and analyze the data, and 3) share invaluable malaria research data and results to help achieve malaria elimination.

## ICEMR Data Management

### Data collection and data capture.

The ICEMR Program was implemented during a period of rapid advances in electronic data capture (EDC) technologies and methods for remote and low-resource settings. EDC has several potential benefits over data collection onto paper forms including 1) reducing the risk of lost paper records, 2) eliminating the delay to enter data from paper forms, 3) reducing the number of staff required for data entry, 4) enabling immediate data quality checks and preliminary analyses, 5) enabling immediate redundant data backups when entered into enabled databases, and 6) providing state-of-the-art training to field and data staff.

It should also be noted that EDC also poses potential problems such as systematic errors originated in software, risk of failure of electronic devices, and an increased skill set required to run a data operation as compared with paper-based forms. A poll of the ICEMR data core staff revealed common barriers to full implementation of EDC, many of which reflect the fact that ICEMR projects are typically located in low-resource, remote settings throughout the world. These barriers include unreliable electricity, low-bandwidth internet connections, unreliable cellular connections, hardware expense, lack of trained personnel, lack of on-site technical support, language differences, complexity of EDC field training/oversight, and in at least one ICEMR (Colombia) the safety of field operators is compromised by the use of Global Positioning System (GPS)–enabled devices as they could be perceived as military targets by guerrillas. Furthermore, some ICEMRs report that filling out paper forms/log books can be easier and be perceived as faster than using electronic screens or keyboards. All ICEMRs currently use some paper forms for informed consent documentation and as a backup in cases of electricity or cellular outage. Although ICEMR databases are securely backed up locally and/or in distributed web-based environments, electronic data continue to have limits in these research settings. As noted by one ICEMR utilizing EDC and a web-based database, storing data in a web-based database greatly enhances study collaboration; however, data cannot be entered or accessed when data connectivity is disrupted at the field site bringing the data operations core to a halt. The continued development and improved electronic infrastructure (including enhanced cellular and cellular data coverage) at these sites over the remaining years of the ICEMR may further enable or enhance ongoing efforts to streamline data capture methods.

As of 2014, five of 10 ICEMRs have implemented EDC using tablet, cellular phone, or computer hardware, primarily using the Open Data Kit (ODK; opendatakit.org) software to direct the captured data into Research Electronic Data Capture (REDCap), MS Access, or custom databases. The remaining ICEMRs use hybrid methods, combining paper collection and EDC. For example, field data collection where cellular connections are inadequate are manually entered into REDCap databases by data entry staff while laboratory results are directly entered into the databases at the Internet-connected research facilities.

REDCap is a web application in use by a number of ICEMR centers that lets investigators easily design and manage databases for research studies. REDCap features flexible and user-friendly tools for designing online data entry forms, controlling user access, auditing user activity, importing files and data, and exporting data as Excel or PDF files or preformatted for statistical packages such as SPSS (IBM Corp., Armonk, NY) and R (R Development Core Team, Vienna, Austria). It is available free of charge on joining the REDCap Consortium (http://project-redcap.org/), a global network of institutions that use the application. Data can be transcribed from paper forms to online REDCap data entry screens by designated data entry personnel; alternately, REDCap can be paired with ODK software to allow field workers to capture data directly to mobile devices, for subsequent upload to a REDCap database. ODK is an open-source set of tools complementary to REDCap, which focuses on mobile data collection solutions. Data captured via ODK is usually imported into REDCap for consolidation of records.

As an illustration of data collection, the Malawi ICEMR used different procedures at different times and in different studies, according to the needs and capacity of the research. The initial cross-sectional surveys used paper-based data collection as outlined here:

1. Informed consent was collected on paper.
2. Demographic data were collected on paper with sticker barcodes linked to blood samples collected. The paper also recorded interview status, entomological collections, and clinical data including temperature and hemoglobin.
3. Paper logs were kept for all thick smear and polymerase chain reaction (PCR) malaria test results.
4. Paper-based data were entered by a data entry clerk online into REDCap using a laptop or desktop computer. The PCR and thick smear results entered were reviewed independently by another person. This review process is still in the process of being formalized.

Subsequent Malawi ICEMR cross-sectional surveys, case–control studies, and cohort studies used a hybrid data collection method, using paper forms where needed and tablets or laptops to capture the majority of data. Data were transferred using ODK to online and offline REDCap databases. The following example helps to illustrate the complexity of EDC in remote settings:

1. Collect informed consent on paper.
2. EDC using tablets or laptops for all household and individual interviews, screening data, logging of specimen collection, environmental records, entomological forms, GPS coordinates for each household, and laboratory results.
3. Transfer data using ODK into REDCap databases. In some sites with unreliable internet connectivity, data were exported as an encrypted copy to a thumb drive every day and transported by car to the data center for uploading.

### Ethical practices.

Serious consideration must be given to ethical issues when designing and implementing information systems to support research involving human subjects. Research funded by the NIH is subject to laws governing ethical conduct of research, under the oversight of the U.S. Department of Health and Human Services. The conceptual framework to which institutional review boards are bound is rooted in the Belmont Report,^8^,^9^ according to which the driving principles of ethical research are 1) respect for persons (courtesy, respect, and transparency with patients, resulting in informed consent), 2) beneficence (“do no harm”), and 3) justice (reasonable, nonexploitative, and fair conduct of research). This framework is regulated in the United States by the Title 45, Part 46 of the Code of Federal Regulations (45 CFR 46).^10^

The ICEMR Program is implemented in international and often low-income settings. The same safeguards that prevent patient data from being misused are in accordance with the principles of respect, beneficence, and justice that all research funded by U.S. federal dollars must comply with. Furthermore, we believe all human subjects' research should provide a level of protection of patient data to the highest standards possible. However, since most countries in which malaria research is conducted do not have the same level of regulation and enforcement of patient privacy as the United States, we consider that the standards of privacy for the ICEMRs must be equal or superior to those available in the United States.

Although existing regulations provide guidance, they do not provide clear operational guidelines for the implementation and use of information systems in international settings. All ICEMR studies comply with 45 CFR 46, ICH GCP E6, FDA 21 CFR Part 11, NIH Clinical Terms of Award, and International Review Board–approved protocols. Some of the ICEMRs have also adopted relevant portions of the U.S. Health Insurance Portability and Accountability Act of 1996 (HIPAA) to non-U.S. settings as guidelines to operationalizing data protection procedures. Specifically portions of the Privacy and Security Rules of Title II, known as the Administrative Simplification Provisions (ASP) have been adopted. The Privacy Rule establishes regulations for the use and disclosure of Protected Health Information, which includes information about health status, provision of health care, or payment for health care that can be linked to an individual. The ASP Privacy Rule states that research teams must 1) inform individuals about privacy policies, 2) disclose information to individuals on request, 3) provide a mechanism for individuals to make corrections to their information, 4) take reasonable steps to ensure confidentiality of communication, and 5) designate a privacy official and contact information. The Security Rule defines three types of security safeguards: 1) administrative: manuals, roles, training, third parties, contingency, internal audits; 2) physical: hardware and software management, access, facility, security plan, policies for workstation use, third party's physical access; 3) technical: encryption and firewalls, audit trail, data corroboration, authentication, documentation, configuration, and risk management plan.

Once the data in the ICEMRs databases are exported to public repositories, additional safeguards apply. PlasmoDB (described in the Data Sharing/Central Database section below), as part of a National Institute of Allergy and Infectious Diseases (NIAID) Bioinformatics Resource Centers, has to comply with National Institute of Standards and Technology (NIST) SP 800-37, Guide for the Security, Certification and Accreditation of Federal Information Systems and NIST 800-53, Recommended Security Controls for Federal Information Systems.

The regulations cited above for the protection of medical information are specific to the United States, since it is the source of funding for the ICEMR projects, but we acknowledge the existence of additional frameworks such as the 1995 Council of European Union Data Protection Directive that provides clear directives for the capture, processing, and movement of personal data. Developing nations have started to produce similar legislations, for example, India's Information Technologies (Reasonable Practices and Procedures and Sensitive Personal Data or Information) Rules, 2011. This is a welcome trend, as we recognize that regulations devised for high-income countries may not always be the most appropriate for lower income countries. In case of competing regulations, the ICEMR Program has opted to adopt the stricter standard between the country where research is conducted and the country where the research funds are originated.

### Structured procedures.

The success of a project depends absolutely on the integrity and quality of the data captured. Perhaps the most important aspect of data capture is the use of standard operating procedures (SOPs), that is, step-by-step written instructions to complete data acquisition, QC, analysis, exchange, and/or transition.^11^ Ideally every SOP should include 1) definition of inputs, 2) stipulation of execution responsibilities, 3) time frame for execution, and 4) expected outcome. ICEMR projects have developed SOPs that cover all aspects of ICEMR data management including the following:

Privacy and data security: tiered data access, data audit controls, data sharing, HIPAA Privacy Rule, HIPAA Security Rule, GPS data management
Data collection: data collection procedures, smartphone data transfer, development and maintenance of SOPs, case report form design, data dictionary
Sample and data integrity: storage of paper case report forms (CRFs), database changes, barcoding, QC for case report forms, QC for samples, database configuration management, backup and recovery, tablet and laptop management, uploading and synchronizing data with REDCap server, data cleaning, initiating and logging into offline REDCap
Data analysis and reporting: building analysis pipelines, testing analysis pipelines, data exchange, reporting

A collection of representative SOPs showcasing procedures from multiple ICEMRs as well as a complete master data management plan that includes SOPs, architectural considerations, and roles and responsibilities of the personnel required to run a data core have been included in the Supplemental Material.

### Quality assurance and quality control.

The most generic, reusable, and important SOP is the one related to QA and QC. We define QA as a set of processes and safeguards to “prevent” data errors, including consistent checks of data integrity, completeness, and correctness. We define QC as a set of processes intended to “mitigate” or “eliminate” the impact of errors that have occurred during data capture and/or processing and that were not prevented by QA procedures. QC requires periodic tasks (e.g., weekly for data, daily for analysis) conducted throughout the duration of the ICEMR projects. Particularly, QC includes the process of continuous improvement of QA, so that past errors are prevented from recurring.

There are numerous strategies and methods for QC, which one to use depends on the situation at hand. The baseline for QC is error detection. In the simplest case, the error present in a data set is an independent and identically distributed random variable (a SOP for the QC of a random variable is explained in the Supplemental Material). A random QC sample contains an amount of errors proportional to the overall number of errors in the data universe. When errors are found during QC, they are corrected and a new random QC sample is selected without replacement. Every time that this process takes place, the overall rate of error is reduced. Therefore, by repeating the QC process several times, it is possible to reduce the overall error to a threshold of acceptable error (note that the threshold of acceptable error could be zero). This protocol for QC provides a simple and effective method to guarantee that overall errors remain under certain threshold. However, there are special cases that deviate from this simple paradigm, for example, systematic errors because of instruments or operators, which result in error bias. In those cases, a systematic process of QC can be defined in a similar way as it was done here for the general simplest case.

### Software quality attributes.

There is an essential need for quality in the software implementations that support the mission of the ICEMRs. The operation of each ICEMR depends on a reliable and effective set of information systems to manage all aspects of the projects, from accounting to molecular biology. All these systems need to exhibit a series of attributes without which quality of information cannot be guaranteed. These considerations are independent of QA and QC. Among the most important attributes all ICEMR systems have to exhibit are “availability”, that is, the proportion of time a system is in a functioning condition; “scalability,” that is, the ability of a system to handle growing amounts of data and use; “maintainability,” that is, the ability of a system to make future maintenance and adaptability easier; “performance,” that is, the ability of a system to react in usable times; “security,” that is, the ability of a system to evade unauthorized use, breach of confidential information, denial of service attacks, repudiation (transactions performed by users who later deny their actions), and integrity compromise (unauthorized manipulation of data resulting in loss of integrity); “modifiability,” that is, the extent to which software can be modified after its initial deployment; “testability,” that is, the extent to which the systems can be tested for proper function; “traceability,” that is, extent to which functionality in an information system can be traced back to a requirement; “reusability,” that is, extent to which the systems can be redeployed and reused in different contexts.^12^

### Reproducibility.

Replication of results is the cornerstone of science. However, with increasingly complex systems and computational methods, the likelihood of human-introduced errors and/or systematic errors has increased significantly.^13^–^15^ Among the best practices to ensure reproducibility of results, we particularly considered the following:

Prevent manual data handling: all analyses are scripted to minimize human error by fully automating the pipeline from database to publication quality graphics and tables. The QA/QC for analysis has a SOP that involves the following script tests to mitigate the risk of systematic errors originated in software development and/or black boxes: 1) unit test: conducted by analysts during development; this tests checks that individual components of an analysis pipeline work as expected. (ii) Integration test: Conducted by analysts at the end of an analysis task; this test checks that individual components of an analysis pipeline do not cause overall failure. (iii) Regression tests: Automated daily test that checks the validity of all analyses; this test checks that analysis pipelines run and produce results as expected even after changes are made.
Archive versions of all tools used: each software tool that we use has multiple versions with differences in functionality, in many cases differences in protocols, and in some cases differences in software bugs. Once an analysis is used in a publication, a snapshot version for all systems used, and all data files produces, and all related source code is archived for possible future reference.
Exercise version control of source code: all source code is versioned with source control systems. The source control systems are then part of incremental backups. Once an analysis script is used in a publication, a branch is created in the source code repository to account for variations in all associated scripts that might be required by review processes, which might cause divergence from ongoing progress in these scripts.
Store intermediate steps: all intermediate files are named with unique identifier that permits identification of the specific analysis to which they belong (including versioning). This facilitates identifying what result files need to be archived.
Provide source code for analysis as part of publications: in every publication in which an analysis script is used, the source code, when appropriate, is submitted as part of supplemental materials.

## ICEMR Metadata

The complexity and heterogeneity of ICEMR data with respect to origin, type, and format necessitates accurate and comprehensive metadata collection. By metadata, we are referring to the aggregate of all the information associated with the sample being collected. For example, time and space attributes (collection date, region, country, GPS coordinates), clinical attributes (health status, pregnancy), and genetic attributes (PCR results, presence/absence of drug resistance mutation) all constitute descriptive metadata associated with a single sample. Well-structured data and consistent representation of metadata are needed for accurate data integration and cross-study analyses. The two main challenges in capturing standardized metadata are 1) knowing what minimum information should be captured for malaria studies, such as age of study subject, species of parasite or vector samples collected, geographic location and time of sample collection, clinical presentation, and types of assays performed and 2) what values and data format should be used for consistent representation, for example, numerical versus alphabetical values, milligram versus microgram measurements.

To address these two issues, representatives of various ICEMR groups collaborated to develop a common minimal data dictionary, which includes a list of metadata fields and attributes. The data dictionary was established by reviewing ICEMR data sources (including case study and collection forms) and identifying the data fields that are important for studying epidemiological patterns of malaria, interaction of host and parasite, and associations of genotype and phenotype. The following broad categories were identified as important for guiding the capture of metadata:

Study type
General information of study subject, such as age, gender
Sample collection date and location
Relevant clinical phenotypes such as fever and other symptoms
Parasite detection assays and results
Molecular assays and results

Name, description, allowed values, and data format for each metadata field were specified in the data dictionary.

**Figure 2.**
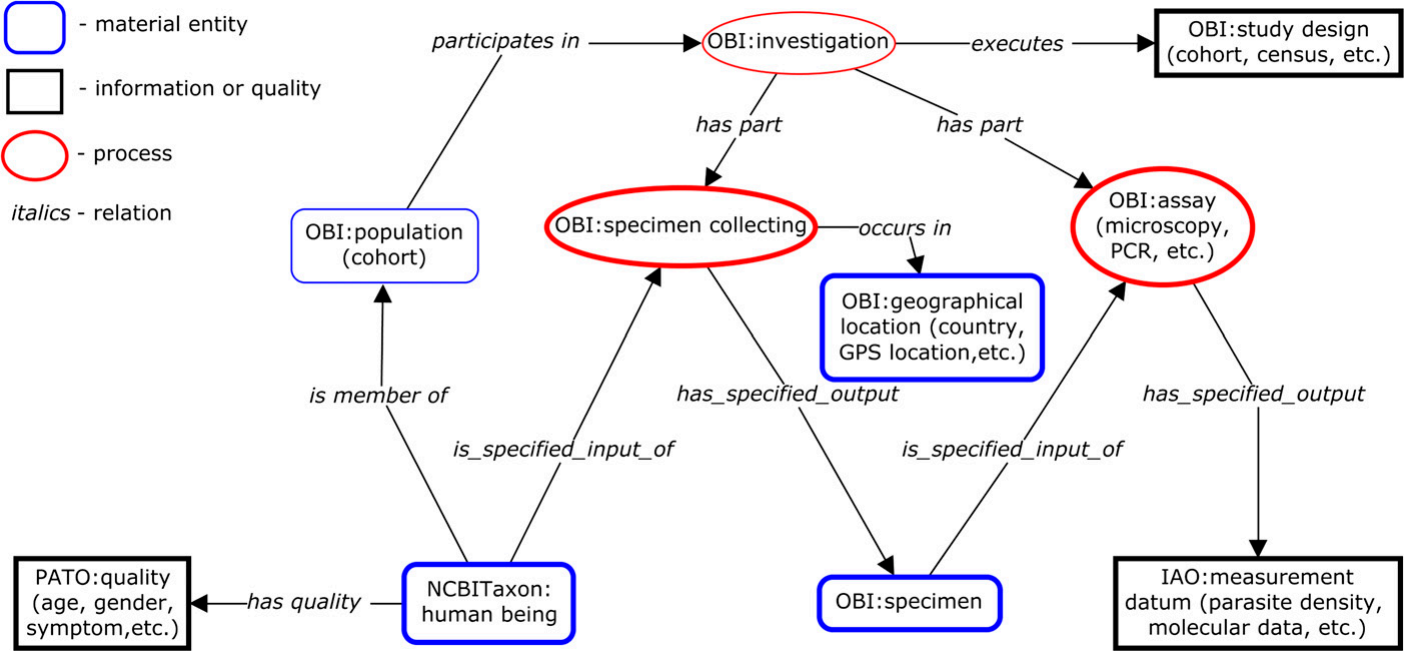
Ontology-based representation of the International Centers of Excellence for Malaria Research (ICEMR) metadata. The data collected in the minimal ICEMR submission form are in thick bordered shapes. Metadata are classified into material (round-cornered rectangles), information or quality (rectangle), or process (oval) entity. Italicized text represents relations. The ontology terms are indicated by using ontology name abbreviations as a prefix: IAO (Information Artifact Ontology), OBI (Ontology for Biomedical Investigations), PATO (Phenotypic quality Ontology), NCBITaxon (NCBI organismal classification).

Recognizing that each ICEMR will have study-specific terminology and metadata, we relied on established ontologies (a set of defined terms and relations between them) to support consistent representation of data for cross-ICEMR data analysis. Specifically, we used the Ontology for Biomedical Investigations (OBI), which is one of eight official components of the Open Biological and Biomedical Ontologies (OBO) Foundry (http://www.obofoundry.org/). The OBO Foundry provides a set of interoperable ontologies in biological and biomedical domains, which were developed following a common design and philosophy using a common upper level ontology and a common set of relationships.^16^ OBI covers all aspects of a biological and clinical investigation including design of an investigation, biomaterials, instruments and protocols used, data generated, and type of analysis applied to the data.^17^ OBI was used as the underlying semantic framework since it can represent all aspects of an investigation and has been used by other metadata standard.^18^ By mapping the minimal ICEMR metadata to OBI/OBO terms, we were able to represent metadata in a standard manner and reveal relations between metadata fields (Figure 2

). The top section of Figure 2 illustrates the type of investigation, such as a cohort or a census study design. The middle section reveals that the investigation has two parts: specimen collecting and assay. The specimen collection occurs in a specific geographic location (such as country, or shown as GPS data) and has inputs and outputs. The input is the human who has qualities such as age, sex, or symptoms (lower left section). The output of specimen collecting is the specimen itself which in turn can be an input of downstream assays (lower right).

Semantic representation of metadata allows us to easily evaluate whether the data dictionary captures all the important metadata needed for the intended use. In addition, the use of ontologies enables harmonization between ICEMR metadata and those from other standards in particular the NIAID/Genome Sequencing Centers for Infectious Diseases (GSCID)/Bioinformatics Resource Centers (BRC) standard.^18^ Using a common framework enables effective data comparison between diverse groups and regions and facilitates data integration from/to other resources. The ICEMR metadata cover all required metadata specified in the NIAID/GSCD/BRC Sample Application Standard.

To facilitate ICEMR data submission and incorporation into PlasmoDB, a metadata collection form (Excel format) (Supplemental Table 1) was created based on the data dictionary. Descriptions of each metadata field were added in the submission form including information about the allowed values (including ontology terms) for any given field.

## Data Sharing/Central Database

The NIH expects the timely release and sharing of final research data from NIH-supported studies for use by other researchers, as specified in the NIH Statement on Sharing Research Data (NOT-OD-03-032). Investigators submitting an NIH application seeking $500,000 or more in direct costs in any single year are expected to include a plan for data sharing. Each of the 10 ICEMRs has submitted a Data Sharing Plan that was approved before funding. The time frame for release and sharing of data is no later than the acceptance for publication of the main findings from the final data set. As the ICEMRs are ongoing, not all the data produced has been made available yet; however, the collaboration with PlasmoDB was designed expressly to encourage and facilitate sharing not only across the ICEMRs but with the wider research community.

**Figure 3.**
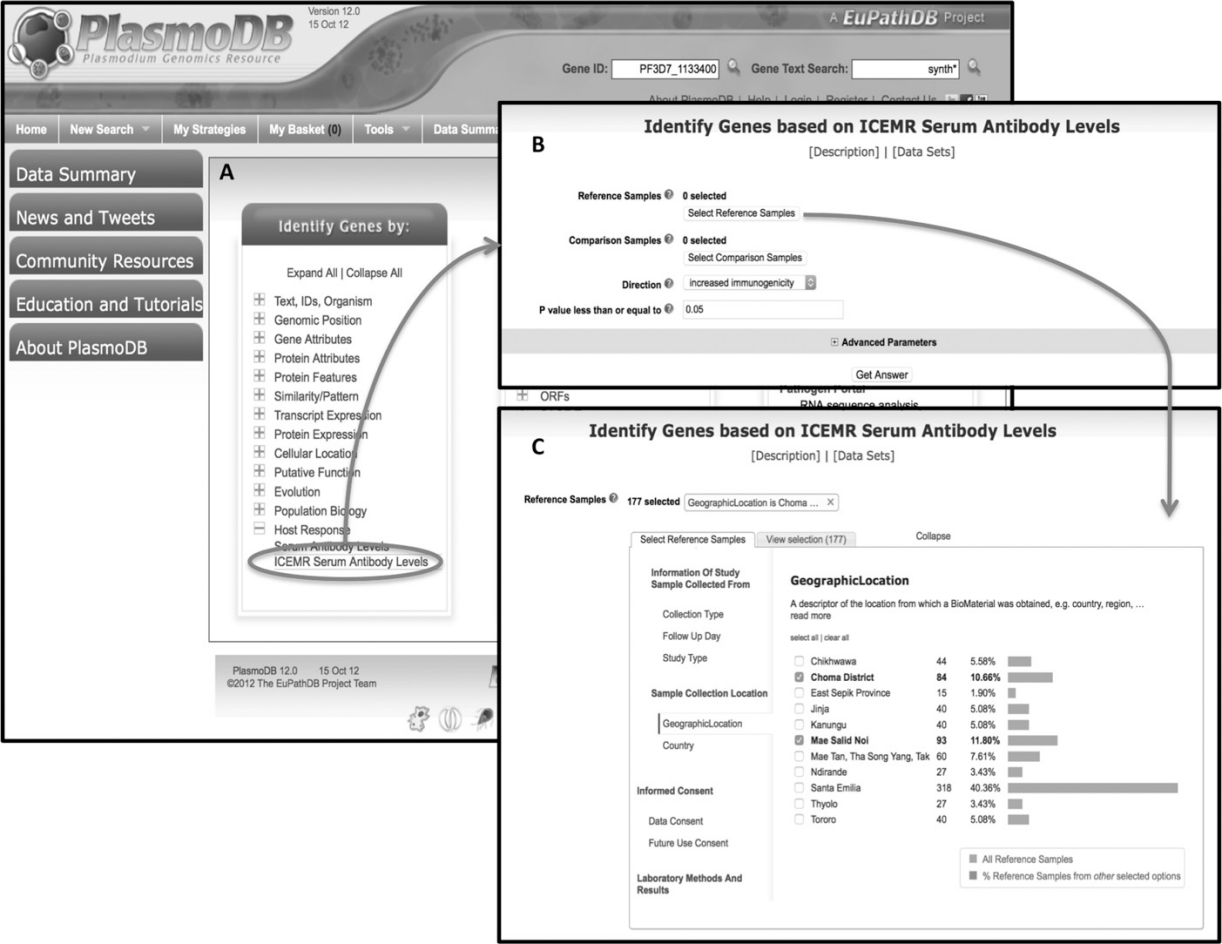
Screenshots from the International Centers of Excellence for Malaria Research (ICEMR)–specific PlasmoDB site. (**A**) Access to the antibody array data is achieved by clicking on the “ICEMR Serum Antibody Levels” link under the host response category on the PlasmoDB homepage. (**B**) The antibody levels form allows the selection of samples to compare with each other based on their metadata. (**C**) The geographic location filter allows selection of samples based on their geographic origin.

**Figure 4.**
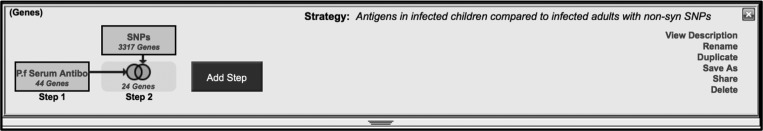
Screenshot from the current production PlasmoDB site depicting a search strategy that combines antibody array data results with population data. Step 1 of the strategy defines genes (antigens) from infected children compared with infected adults.^19^ In step 2, results from step 1 are intersected with a search that identifies all genes that contain genes with a at least five non-synonymous polymorphisms. This search may be accessed at http://plasmodb.org/plasmo/im.do?s=2d611e1195d75ca7.

To facilitate mining of data generated by the ICEMRs, PlasmoDB has developed an ICEMR-specific web interface that enables ICEMR researchers to query their data. Since collected metadata have been standardized as described above, ICEMR samples can be filtered and compared based on the available metadata. Such filtering and comparison can be done on samples from an individual ICEMR or from across the 10 ICEMRs. Experimental results available for the filtered samples can then be analyzed. As a first iteration, antibody array data from serum samples have been integrated into this database. There are several advantages to this approach:

1. Filtering and comparing samples based on associated metadata such as disease status, age, geographic location, enables both inter- and intra-ICMER analyses (Figure 3).
2. The intuitive graphical user interface available on PlasmoDB allows for the identification of sets of results that are common between or unique to specific ICEMRs. For example, reactive antigens from samples from infected adults can be compared with samples from uninfected adults from the Amazonia ICEMR, allowing one to define the set of antigens that are either unique or in common with the east African ICEMR (Supplemental Figure 1).
3. The interface provides a visual and graphical mechanism for reviewing the data for QA/QC.
4. ICEMR data can be searched in the context of all other data available in PlasmoDB, including expression data (transcriptomic and proteomic), population diversity data, metabolic pathways, and so on. This process also enables the ICEMR projects to access the larger Bioinformatics Resource Center mechanisms. For example, searches in PlasmoDB can be used to identify genes (antigens) with increased immunogenicity based on available metadata followed by defining the subset of these genes that are under diversifying selection (containing a high number of non-synonymous mutations) based on isolate sequencing data (Figure 4).

## Discussion

Data management is a common cornerstone across ICEMR projects. The role of data managers goes beyond that of simple technology operators; it is a role more akin to a chief technology officer, that is, an actor that can provide valuable information to make strategic decisions that affect the success of a project. Data management has to be taken into consideration before data capture, during execution of protocols and during analysis and communication of results.

Identification of data commonalities and associated mapped ontologies among the diverse settings and research data types contained within the spectrum of ICEMR site investigations presents significant opportunities for “in-silico” exploratory research. Cross-ICEMR comparisons of identified statistical associations can add to the significance of scientific findings through evidence demonstrating reproducibility of results in other geographic settings, or by allowing agglomeration of data for use in meta-analysis techniques. Examination of emergent properties or effect modification or interaction between settings or subject characteristics that differ at the level of geographic sites become possible with aggregation of related structured data across the global settings contained within the 10 ICEMR sites.

Perhaps the most important aspect of data management is to ensure the quality and consistency of data that are used in analysis and subsequently reported to broad audiences (scientific community, government, and practitioners). To achieve this desired level of quality, analyses have to be integrated with the procedures of data management, which is conducive to having automated pipelines for analysis. This, however, requires the buy-in from principal investigators and researchers consuming data. A common situation occurs when researchers ask data managers to provide data to conduct analyses that involve massaging data and unscripted actions (i.e., use of menus in analysis software packages or manual file alterations); this is problematic because data corrections as a result of QA/QC procedures are common, and manual steps have to be reproduced at every change. Automated pipelines of analysis involve the input of the researchers, mediated by the quality provided by data managers. We have implemented the ideal case in which analyses conducted in PlasmoDB are shared strategies that support reproducible science through providing the workflow used to generate a result that may be cited in a publication (see Figure 3).

Data sets may be used decades after collection for unforeseen purposes using new technologies. For instance, data collected between 1940 and 1963 at the Central State Hospital (Milledgeville, GA) and the NIH Laboratories (Columbia, SC) representing survival time of patients treated for neurosyphilis with malaria therapy^20^ were used by Johnston and others^21^ to produce a stochastic model of transmission intensity in a community (represented by *R*_0_) from mechanistic within-host principles. This use of data more than five decades after collection was not foreseen by the patients or their caregivers. Even though this particular data set predates informed consent, we can anticipate future analogous broad use of data being collected today; this is because that there are no statutory rules regarding the length of informed consent. Given the potential benefit of posterior analysis of current data sets, some effort should be invested in ensuring that data preservation allows such analyses in the future. Ethical considerations in this regard are not a settled issue and are a clear area of future work; ICEMRs have prepared for that future by providing the highest standard available today to patient's data protection.

Significant investigatory opportunities arise from linkage of multilevel classes of data contained within the ICEMR investigations that range from environmental measures of climate, hydrology, and elevation to population characteristics, residential characteristics, person-level exposures, mosquito vector data down to serologic and molecular level data of hosts, vectors, and parasites. Putative relationships and causal processes can be explored over longitudinal datasets utilizing such data without the significant costs of designing and implementing studies specific to those questions. Of course, this is true of any significant research database, but what makes the cross-ICEMR databases unique is the breadth and depth of the data at a global level. The utility of such databases to a broad range of scientists is subject to its availability, ease of use, and the tools that researchers use to access the data and perform these exploratory analyses. Efforts are currently underway to integrate ICEMR data and analysis into public interfaces such as PlasmoDB and GenBank, such that they will be accessible to the research community at large; these efforts include considerations to share source code and data dictionaries.

There are other efforts in the world of malaria research that share commonalities with the ICEMR projects. At the cellular and molecular level, MalarImDB^22^ offers aggregated information about immunology and pathogenesis. At the epidemiological level, the INDEPTH Network^23^ offers patterns for health and demographic surveillance systems across many sites in low- and middle-income countries; similarly, the Demographic and Health Survey^24^ offers comparable nationally representative household surveys from more than 85 countries. The link between molecular and epidemiological data is bridged by the Malaria Genomic Epidemiology Network^25^, a consortium of researchers from 21 countries addressing large-scale studies of genomic variation. Mapping and visualization of global patterns of malaria is currently the focus of the Malaria Atlas Project^26^. This list is not exhaustive, but it is representative of the data repositories available in the world of malaria research. The integration of the ICMER data and other research initiatives is clear direction for future research.

The federated data management operation mounted by the ICEMR Program provides an example of a solid foundation on which malaria elimination can be achieved. In addition, the lessons learned and methods developed for data management during the course of the ICEMR projects are of ample applicability, thus the benefit of this aggregated knowledge expands beyond the field of malaria and outside the realm of research.

## Supplementary Material

Supplemental Data.

## References

[1] Schadt EE, Linderman MD, Sorenson J, Lee L, Nolan GP (2010). Computational solutions to large-scale data management and analysis. Nat Rev Genet.

[2] Rao M (2012). The International Centers of Excellence for Malaria Research. Acta Trop.

[3] Wright A, Sittig DF (2008). SANDS: a service-oriented architecture for clinical decision support in a National Health Information Network. J Biomed Inform.

[4] Kashyap V, Morales A, Hongsermeier T (2006). On implementing clinical decision support: achieving scalability and maintainability by combining business rules and ontologies. AMIA Annu Symp Proc.

[5] Megy K, Emrich SJ, Lawson D, Campbell D, Dialynas E, Hughes DS, Koscielny G, Louis C, Maccallum RM, Redmond SN, Sheehan A, Topalis P, Wilson D, VectorBase Consortium (2012). VectorBase: improvements to a bioinformatics resource for invertebrate vector genomics. Nucleic Acids Res.

[6] Aurrecoechea C, Brestelli J, Brunk BP, Dommer J, Fischer S, Gajria B, Gao X, Gingle A, Grant G, Harb OS, Heiges M, Innamorato F, Iodice J, Kissinger JC, Kraemer E, Li W, Miller JA, Nayak V, Pennington C, Pinney DF, Roos DS, Ross C, Stoeckert CJ, Treatman C, Wang H (2008). PlasmoDB: a functional genomic database for malaria parasites. Nucleic Acids Res.

[7] Bauer S (2008). Mining data, gathering variables and recombining information: the flexible architecture of epidemiological studies. Stud Hist Philos Biol Biomed Sci.

[8] Cassell EJ (2000). The principles of the Belmont report revisited: how have respect for persons, beneficence, and justice been applied to clinical medicine?. Hastings Cent Rep.

[9] Vollmer SH, Howard G (2010). Statistical power, the Belmont report, and the ethics of clinical trials. Sci Eng Ethics.

[10] Gallin JI, Ognibene FP (2012). Principles and Practice of Clinical Research.

[11] Marcus DS, Harms MP, Snyder AZ, Jenkinson M, Wilson JA, Glasser MF, Barch DM, Archie KA, Burgess GC, Ramaratnam M, Hodge M, Horton W, Herrick R, Olsen T, McKay M, House M, Hileman M, Reid E, Harwell J, Coalson T, Schindler J, Elam JS, Curtiss SW, Van Essen DC (2013). Human Connectome Project informatics: quality control, database services, and data visualization. Neuroimage.

[12] Gomaa H (2011). Software Modeling and Design: UML, Use Cases, Patterns, and Software Architectures.

[13] Prlić A, Procter JB (2012). Ten simple rules for the open development of scientific software. PLoS Comput Biol.

[14] Sandve GK, Nekrutenko A, Taylor J, Hovig E (2013). Ten simple rules for reproducible computational research. PLoS Comput Biol.

[15] Osborne JM, Bernabeu MO, Bruna M, Calderhead B, Cooper J, Dalchau N, Deane C (2014). Ten simple rules for effective computational research. PLoS Comput Biol.

[16] Smith B, Ashburner M, Rosse C, Bard J, Bug W, Ceusters W, Goldberg LJ, Eilbeck K, Ireland A, Mungall CJ, Leontis N, Rocca-Serra P, Ruttenberg A, Sansone SA, Scheuermann RH, Shah N, Whetzel PL, Lewis S, Consortium OBI (2007). The OBO foundry: coordinated evolution of ontologies to support biomedical data integration. Nat Biotechnol.

[17] Brinkman RR, Courtot M, Derom D, Fostel JM, He Y, Lord P, Malone J, Parkinson H, Peters B, Rocca-Serra P, Ruttenberg A, Sansone SA, Soldatova LN, Stoeckert CJ, Turner JA, Zheng J, OBI consortium (2010). Modeling biomedical experimental processes with OBI. J Biomed Semantics.

[18] Dugan VG, Emrich SJ, Giraldo-Calderón GI, Harb OS, Newman RM, Pickett BE, Schriml LM, Stockwell TB, Stoeckert CJ, Sullivan DE, Singh I, Ward DV, Yao A, Zheng J, Barrett T, Birren B, Brinkac L, Bruno VM, Caler E, Chapman S, Collins FH, Cuomo CA, Di Francesco V, Durkin S, Eppinger M, Feldgarden M, Fraser C, Fricke WF, Giovanni M, Henn MR, Hine E, Hotopp JD, Karsch-Mizrachi I, Kissinger JC, Lee EM, Mathur P, Mongodin EF, Murphy CI, Myers G, Neafsey DE, Nelson KE, Nierman WC, Puzak J, Rasko D, Roos DS, Sadzewicz L, Silva JC, Sobral B, Squires RB, Stevens RL, Tallon L, Tettelin H, Wentworth D, White O, Will R, Wortman J, Zhang Y, Scheuermann RH (2014). Standardized metadata for human pathogen/vector genomic sequences. PLoS One.

[19] Crompton PD, Kayala MA, Traore B, Kayentao K, Ongoiba A, Weiss GE, Molina DM, Burk CR, Waisberg M, Jasinskas A, Tan X, Doumbo S, Doumtabe D, Kone Y, Narum DL, Liang X, Doumbo OK, Miller LH, Doolan DL, Baldi P, Felgner PL, Pierce SK (2010). A prospective analysis of the Ab response to *Plasmodium falciparum* before and after a malaria season by protein microarray. Proc Natl Acad Sci USA.

[20] Sama W, Dietz K, Smith T (2006). Distribution of survival times of deliberate *Plasmodium falciparum* infections in tertiary syphilis patients. Trans R Soc Trop Med Hyg.

[21] Johnston GL, Smith DL, Fidock DA (2013). Malaria's missing number: calculating the human component of R0 by a within-host mechanistic model of *Plasmodium falciparum* infection and transmission. PLoS Comput Biol.

[22] Deroost K, Opdenakker G, Van den Steen PE (2014). MalarImDB: an open-access literature-based malaria immunology database. Trends Parasitol.

[23] Sankoh O, Byass P (2012). The INDEPTH Network: filling vital gaps in global epidemiology. Int J Epidemiol.

[24] Corsi DJ, Neuman M, Finlay JE, Subramanian SV (2012). Demographic and health surveys: a profile. Int J Epidemiol.

[25] Achidi EA, Agbenyega T, Allen S, Amodu O, Bojang K, Conway D, Williams T (2008). A global network for investigating the genomic epidemiology of malaria. Nature.

[26] Moyes CL, Temperley WH, Henry AJ, Burgert C, Hay SI (2013). Providing open access data online to advance malaria research and control. Malar J.

