# Supplementary material for: A Framework for Global Collaborative Data Management for Malaria Research
Source: Am J Trop Med Hyg. 2015 Sep 2;93(3 Suppl):124–32. doi: 10.4269/ajtmh.15-0003 (PMC4574270; doi:10.4269/ajtmh.15-0003)
Supplement: Supplementary file 1 [file SD2.pdf]

Supplemental Material for
A Framework for Global Collaborative Data Management for Malaria Research

Juan B. Gutierrez*, Omar S. Harb, Jie Zheng, Daniel J. Tisch, Edwin D. Charlebois, Christian J. Stoeckert Jr., Steven A. Sullivan

Table of Contents

Computer System Access Request Form 2
PROGRAM FOR RESISTANCE, IMMUNOLOGY, SURVIELLANCE, AND MODELLING OF MALARIA PROJECT (PRISM)

Change Request Form 3
PROGRAM FOR RESISTANCE, IMMUNOLOGY, SURVIELLANCE, AND MODELLING OF MALARIA PROJECT (PRISM)

Standard Operating Procedure 5
PRISM DATA-001: GPS DATA MANAGEMENT
PROGRAM FOR RESISTANCE, IMMUNOLOGY, SURVIELLANCE, AND MODELLING OF MALARIA PROJECT (PRISM)

Standard Operating Procedure 9
PRISM DATA-005: GENERATION OF BARCODE LABELS
PROGRAM FOR RESISTANCE, IMMUNOLOGY, SURVIELLANCE, AND MODELLING OF MALARIA PROJECT (PRISM)

Standard Operating Procedure 13
PRISM DATA-010: COMPUTER SYSTEM SECURITY
PROGRAM FOR RESISTANCE, IMMUNOLOGY, SURVIELLANCE, AND MODELLING OF MALARIA PROJECT (PRISM)

Standard Operating Procedure 16
PRISM DATA-011: SYSTEM BACKUP, RECOVERY AND CONTINGENCY PLANS
PROGRAM FOR RESISTANCE, IMMUNOLOGY, SURVIELLANCE, AND MODELLING OF MALARIA PROJECT (PRISM)

Standard Operating Procedure 21
PRISM DATA-012: CHANGE CONTROL PROCEDURE
PROGRAM FOR RESISTANCE, IMMUNOLOGY, SURVIELLANCE, AND MODELLING OF MALARIA PROJECT (PRISM)

Standard Operating Procedure PRISM ENTO - Collection of indoor mosquitoes using CDC light traps and sample processing in the PRISM study 24
PROGRAM FOR RESISTANCE, IMMUNOLOGY, SURVIELLANCE, AND MODELLING OF MALARIA PROJECT (PRISM)

Human Genetics Protocol #5 - How to Assign IDs for the Human Genetics Community Survey 32
MALARIA EVOLUTION IN SOUTH ASIA RESEARCH CENTER

Data Access Protocol #2 - Access to MESA Electronic Databases 36
MALARIA EVOLUTION IN SOUTH ASIA RESEARCH CENTER

Data Access Protocol #1 - Storage of MESA Paper Forms and Lab Notebooks 38
MALARIA EVOLUTION IN SOUTH ASIA RESEARCH CENTER

Standard Operating Procedure - Computer Backups 43
PNG Institute of Medical Research - Southwest Pacific ICEMR

Lab Report Batch Form Workflow 46
CSCMI

Master Data Management Plan 49
ICEMR - Non-Amazonian Regions of Latin America

THE REPUBLIC OF UGANDA

PROGRAM FOR RESISTANCE, IMMUNOLOGY, SURVIELLANCE, AND MODELLING OF MALARIA PROJECT (PRISM)

Form 10.1 Computer System Access Request Form

Data Department

This form must be filled out by any staff member who needs access to any computer system used for data management/entry/transfer within the PRISM study. It must be signed by the requester and the Project Investigator/Study Coordinator.

This section filled out by staff member requiring access

Name: ______________________________________

Signature: ___________________________________ Date:___________________

IDRC Data Access Terms and Conditions

You agree to:

1. *Make no attempts to identify study participants.*
2. *Not to transfer data to any third party.*
3. *Not to allow others to use your username and password to access the computer.*
4. *Report immediately to IDRC any disclosure of study participant identity as well as any discovery of flaws or errors in the data.*

This section filled out by Project Investigator/Study Coordinator's

Name: ______________________________________

Signature: ___________________________________ Date:___________________

This section to be filled out by the Data Manger

**Site:**	
**Computer Name:**	
**Username:**	
**Access Details:** *Specify as much detail as possible – user access level, which databases user has access to, level of access, etc.*	
**Effective Date:**	
**Data Manager's Signature:**	
**Date user removed:**	

THE REPUBLIC OF UGANDA

PROGRAM FOR RESISTANCE, IMMUNOLOGY, SURVIELLANCE, AND MODELLING OF MALARIA PROJECT (PRISM)

Form 12.1 Change Request Form

Data Department

Project: □ Cohort □ Community Survey □ School Survey □ Entomology

Form/CRF Name: ____________________________

Old Version #: ________________ **New Version #:** ________________

Completed by: ________________________ **Effective Date:** _____ ________________

**Question #**	**Variable name** *(if new variable)*	**Description of Change**

THE REPUBLIC OF UGANDA

This page is for Data Center use.

**Changes Required**	**Status**	**Completed By**
Update MS Excel Data Dictionary		
Update SQL Server tbldd		
Modify table in MS Access Work database		
Modify Form in MS Access Work database		
Modify table in MS Access Archive database		
Modify table in SQL Server database		
Modify DTS/SSIS		
Copy a new version of Form to website		
Modify tblForms with latest version information		
Recreate DTS(‘s) that exports data		
Recreate Update Triggers		
Recreate Delete Triggers		
Recreate LastMod Triggers		
Update batch file for zipping		
Modify the rights on newly created tables (editor/viewer)		

THE REPUBLIC OF UGANDA

UCSF

PROGRAM FOR RESISTANCE, IMMUNOLOGY, SURVIELLANCE, AND MODELLING OF MALARIA PROJECT (PRISM)

Standard Operating Procedure

PRISM DATA-001: GPS DATA MANAGEMENT

**SOP ID#: PRISM DATA-001**	**Version 1.0**			**DIVISION: DATA**
Written date: 31 December 2010<br>Effective date: 1 January 2011<br>SOP review cycle: N/A<br>SOP reviewers: N/A				Written by: Sarah Staedke, Geoff Lavoy, Ruth Kigozi<br>Approved by: Joan Nankabirwa
**Vers#**	**Date Modified:**	**Modified by:**	**Approved by:**	**Approved Signature:**
**Vers#**	**Date Reviewed:**	**Annual Review (no changes required) by:**		**Reviewed Signature:**

Target Audience:

Principal Investigator		Data Manager	X
Investigators	X	Data Officer	
Study coordinators	X	Lab staff	
Data Center Director	X	Local Assistants	

The following study personnel have read and understood SOP#: PRISM DATA-001:

Date	Name	Signature

THE REPUBLIC OF UGANDA

SOP ID#: PRISM DATA-001	DIVISION: DATA
Effective date: 1 January 2011	Written by: Sarah Staedke, Geoff Lavoy, Ruth Kigozi

I. Title: GPS Data Management

II. Purpose:

To describe the procedures for handling data collected using Global Positioning System (GPS) receivers during the enumeration and mapping of households and schools in Jinja and Kanungu districts, Uganda.

III. Background/Rationale:

We plan to create a map of households, primary schools and other key features in Walukuba sub-county, Jinja district and Kihihi sub-county, Kanungu district using the global positioning system (GPS). GPS uses a constellation of 24-32 satellites that orbit the Earth and transmit precise radio wave signals, allowing GPS receivers to determine their current location. We will use GPS receivers to record the location of all households, primary schools and boundaries of the sub-counties, parishes, and villages, and locations of other key features such as health centers, drug shops, and geographic features. The GPS co-ordinates will be captured in the GPS receivers and stored in the GPS database. This SOP provides guidelines and recommendations on how the study personnel should manage the GPS data, including the transfer of the data to a computerized database.

IV. Supplies and Materials

- Garmin eTrex Legend H GPS receiver
- Trip and Waypoint Manager software
- Desktop/ Laptop Computer
- Custom Visual Basic program (named 'GPS Transfer')

V. Definitions:

- **GPS:** Global Positioning System

- **Feature:** A point of interest for which GPS data points can be recorded. Features are made up of positional data points and may be of several types, including point features, line features, and area features, each of which uses different rules for storing and displaying data point. A feature may have several attributes (see below).

- **Waypoint:** A waypoint is a specified geographical location, spot or destination defined by longitude and latitude used for navigational purposes. It is used in the definition of routes and terminal segments.

VI. Procedures

GPS receivers will be used to record the location of all households, primary schools and boundaries of the sub-counties, parishes, and villages, and locations of other key features such as health centers, drug shops, and geographic features. The GPS co-ordinates will be captured in the GPS receivers and stored in the GPS database. Please refer to *SOP PRISM FIELD-001 USING GPS TO MAP AND LOCATE HOUSEHOLDS AND KEY FEATURES* for additional information on using the GPS receivers and labelling key features.

A. Downloading the data from the GPS receivers

- Each day, the data on the GPS receivers should be downloaded to the central computer using the Trip and Waypoint Manager software.
- The software will download the GPS data into a text file, and each file should be labelled with the GPS receiver number + the dates that the data were collected. For example, for data collected using GPS 01 for the week of the 19th of Jan through the 23rd of Jan 2010, the file should be labelled 'GPS01_19012010_23012010')
- Each GPS will have a folder where the text files will be saved. This folder will reside in the C:\GPSdata\RawGPSdata directory. For example, the files from GPS01 will be saved in the following folder: C:\GPSdata\RawGPSdata\GPS01
- The naming convention and location of the files are very important in order for the transfer program to work properly. Dates must consist of 8 characters. For example, the date March 8, 2010 will be saved as 08032010.

B. Transferring the data from the Text file to a MS Access database

- A custom Visual Basic program is used to transfer the data from the text files to a MS Access database. The name of the program is 'GPS Transfer' and will be installed on the computer where the text files are saved.
- The Visual Basic program requires that the MS Access database be in the folder: C:\GPSdata and be named: GPS_Data.mdb
- To transfer the files, run the program 'GPS Transfer' and click the 'Transfer Data' button.

C. Backing-up the GPS database

- Each day the MS Access database with the GPS data (C:\GPSdata\GPS_Data.mdb) will be copied to an external hard drive with the naming convention of GPS_Data_*day*.mdb. For example, for the day; January 20, 2010, the database will be named GPS_Data_20JAN2010.mdb

- The file will then be transferred to the server in Mulago, Kampala and copied to D:\PRISM\EnumerationSurvey\GPS_Data. This should occur at least once a week.

- All data on the server is backed up on a daily basis as per the Data Management System Design Document.

VII. Attachments

- Data Management System Design Document Version 2.0.

VIII. Documentation

- PRISM Protocol, "Enumeration and mapping of households and schools in Jinja and Kanungu districts, Uganda", DMID Protocol Number: 10-0027
- PRISM HHE FIELD–001: Using GPS to map and locate households and key features

PROGRAM FOR RESISTANCE, IMMUNOLOGY, SURVIELLANCE, AND MODELLING OF MALARIA PROJECT (PRISM)

Standard Operating Procedure

PRISM DATA-005: GENERATION OF BARCODE LABELS

**SOP ID#: PRISM DATA-005 Version 1.0**	**DIVISION: DATA**
Written date: 15 May 2011<br>Effective date: 1 June 2011<br>SOP review cycle: Yearly<br>SOP reviewers: Investigators, Data Management Staff	Written by: Geoff Lavoy, Todd Hayes-Birchler<br>Approved by: Emmanuel Arinaitwe

**Vers#**	**Date Modified:**	**Modified by:**	**Approved by:**	**Approved Signature:**

**Vers#**	**Date Reviewed:**	**Annual Review (no changes required) by:**	**Reviewed Signature:**

Target Audience:

Principal Investigator		Data Manager	x
Investigators	x	Data Officer	
Study coordinators	x	Lab staff	
Data Center Director	x	Local Assistants	

The following study personnel have read and understood SOP#: PRISM DATA-005:

Date	Name	Signature

SOP ID#: PRISM DATA-005	DIVISION: DATA
Effective date: 1 June 2011	Written by: Geoff Lavoy, Todd Hayes-Birchler

I. Title: Generation of barcode labels

II. Purpose:

This document describes the procedure for generating barcode labels for sample tracking in the Cohort studies, Cross-sectional surveys in communities and schools, and the Entomology studies.

III. Background/Rationale:

Several samples will be taken throughout the PRISM activities and each sample will have to be tracked individually and be able to be linked back to the original subject and/or household. Barcodes will be used for tracking RDT's as well as tracking storage of blood samples on filter paper, slides, and cryovials. Barcodes will also be used to track mosquitoes.

IV. Supplies and Materials

- MS Access database
- NiceLabel software (Version 5 or later)
- Labels of various sizes (for RDT's, slides, cryovials, and filter paper. ELIZA plates).

V. Procedures

A) Barcode Composition

i) Community surveys and Cohort studies

Each blood sample taken in both the Community surveys and the Cohort studies will have to be identified with a barcode that links the sample back to the Subject ID. The barcode will take the following format: **ABC-XXXX** where:

A: Type of study
B: District
C: Sample type
XXXX: 4 digit random alpha-numeric code (unique)

Note: To avoid confusion, the 4 digit alpha-numeric code will not contain 1, I, 0, O.

The following table shows the codes for Type of Study, District and Sample type.

Type of Study	District	Sample Type
C : Cohorts	K : Kanungu	1 : Thick blood smear
H : Household Survey	J : Jinja	2 : Thin blood smear
S : School Survey	T : Tororo	3 : Plasma
F: Cohort Survey		4 : Filter paper
		5 : RDT

For example a Thin blood smear from the Tororo cohort study would be something similar to:
CT2-SK4E

ii) Entomology Studies

Mosquitoes collected throughtout the Entomology studies will be stored on 96 well ELISA plates. Each ELISA plate will be have a barcode that links it back to the Household, date, and method of colleection. The barcode will take the following format: **AB-XXXX** where:

A: District
B: Collection Method
XXXX: 4 digit sequential numeric number beginning at 0001

The following table shows the codes for District and Collection Method.

District	Collection Method
K : Kanungu	1 : Light Traps
J : Jinja	2 : Human Landing Catches
T : Tororo	3 : Mosquito Abdominal Status

For example an ELISA plate from Kanungu with mosquitoes from a Human Landing Catch would be something similar to:
K2-0001

B) Barcode Printing

There will be 4 types of labels used for the blood samples and ELIZA plates.

1. Slide Labels (for thin and thick blood smears)
2. Cryovial Labels (for Plasma samples)
3. Filter paper labels
4. RDT labels
5. ELIZA

Each type of label will have a unique design using the NiceLabel software and be printed on different sizes of label stock. An MS Access database will house all the necessary barcodes for each type of

sample and will link directly to the NiceLabel software. Labels for a specific Study/Site/Sample can then be pre-printed and sent to the site for use.

Once a label has been printed, it will be deleted from the MS Access database so that there is no chance that the same barcode can be used again.

VI. Documentation

- PRISM Protocol, "Cohort studies to estimate malaria incidence and morbidity in three different epidemiological settings in Uganda", DMID Protocol Number: 10-0063
- PRISM Protocol, "Surveillance of malaria morbidity and coverage of key control interventions: Cross-sectional surveys in communities and schools in three different epidemiological settings in Uganda", DMID Protocol Number: 10-0062
- PRISM Protocol, "Entomological surveillance for estimating malaria transmission intensity in 3 sites in Uganda", DMID Protocol Number: 10-0064

THE REPUBLIC OF UGANDA

UCSF

PROGRAM FOR RESISTANCE, IMMUNOLOGY, SURVIELLANCE, AND MODELLING OF MALARIA PROJECT (PRISM)

Standard Operating Procedure

PRISM DATA-010: COMPUTER SYSTEM SECURITY

**SOP ID#: PRISM DATA-010**	**Version 1.0**			**DIVISION: DATA**
Written date: 20 June 2011<br>Effective date: 01 July 2011<br>SOP review cycle: Yearly<br>SOP reviewers: Data Management Staff				Written by: Geoff Lavoy<br>Approved by: Edwin Charlebois
**Vers#**	**Date Modified:**	**Modified by:**	**Approved by:**	**Approved Signature:**
**Vers#**	**Date Reviewed:**	**Annual Review (no changes required) by:**		**Reviewed Signature:**

Target Audience:

Principal Investigator		Data Manager	X
Investigators	X	Data Officer	X
Study coordinators	X	Lab staff	X
Data Center Director	X	Local Assistants	

The following study personnel have read and understood SOP#: PRISM DATA-010:

Date	Name	Signature

SOP ID#: PRISM DATA-010	DIVISION: DATA
Effective date: 01 July 2011	Written by: Geoff LAvoy

I. Title: Computer System Security

II. Purpose:

To describe the procedures for security surrounding computer systems used in the PRISM study. The procedures described within this SOP are designed to prevent unauthorized access and limit access to the computer systems.

III. Background/Rationale:

This SOP applies to computer systems implemented by the Data Management Center (DMC) which are used for remote data capture (electronic or other), data transmission and data storage and covers computer systems both at remote sites and at the DMC.

This SOP will address physical and logical security.

IV. Responsibility

- The Data Manager is responsible for researching, evaluating and implementing adequate security for all computer systems covered by this SOP.
- The Data Manager is responsible for maintaining security systems and updating security where necessary.
- The Data Manager is responsible for monitoring software vendor sites regularly and installing software vendor fixes (‘patches’) as they become available.
- The Data Manager is responsible for ensuring that data cannot be altered, browsed, queried or reported through software that is not protected by the above mentioned security measures.
- The Data Manager and the Study Coordinator are responsible for providing and updating as necessary, a list of all personnel who have access to the computer system.
- The Data Manager, Study Coordinator, and study personnel at the remote sites and all staff at the DMC are responsible for reporting any breach or potential breach of security of the computer system.

V. Procedures

- The Data Manager will research and evaluate the security measures necessary for each computer system. Following this, security measures will be implemented as appropriate.
- Passwords will be used for access to any computer system.
- Any user who requires access to the system will have to fill out a Computer System Access Request Form (Form 10.1) and have it signed by the Study Coordinator.
- Users will change their password quarterly.

- The Data Manager will establish a relationship with vendors such that Data Management Center is notified of software fixes ('patches') as they are issued. Where a relationship cannot be established, Information Systems will identify a person(s) responsible for and will determine a schedule for monitoring software vendor sites for software fixes ('patches').
- Once a computer system is implemented, the Data Manager and the Project Director will provide a list of personnel who will have access to the computer system and the level of access granted (form 10.1). Access to the System Server(s) will be granted only by the Senior Data Manager or his/her designee. This list will be updated on a regular basis as needed.
- The Data Manager will maintain and update the security measures as necessary.
- When a user no longer needs access to the computer system, the Data Manager will remove the user account and update Form10.1.

THE REPUBLIC OF UGANDA

PROGRAM FOR RESISTANCE, IMMUNOLOGY, SURVIELLANCE, AND MODELLING OF MALARIA PROJECT (PRISM)

Standard Operating Procedure

PRISM DATA-011: SYSTEM BACKUP, RECOVERY AND CONTINGENCY PLANS

**SOP ID#: PRISM DATA-011 Version 1.0**				**DIVISION: DATA**
Written date: 20 June 2011<br>Effective date: 01 July 2011<br>SOP review cycle: Yearly<br>SOP reviewers: Data Management Staff				Written by: Geoff Lavoy<br>Approved by: Edwin Charlebois
**Vers#**	**Date Modified:**	**Modified by:**	**Approved by:**	**Approved Signature:**
**Vers#**	**Date Reviewed:**	**Annual Review (no changes required) by:**		**Reviewed Signature:**

Target Audience:

Principal Investigator		Data Manager	X
Investigators		Data Officer	X
Study coordinators	X	Lab staff	
Data Center Director	X	Local Assistants	

The following study personnel have read and understood SOP#: PRISM DATA-011:

Date	Name	Signature

THE REPUBLIC OF UGANDA

SOP ID#: PRISM DATA-011	DIVISION: DATA
Effective date: 01 July 2011	Written by: Geoff Lavoy

I. Title: System Backup, Recovery and Contingency Plans

II. Purpose:

To describe the procedures for computer system backup and recovery in order to protect the data, systems, and application files from loss. This SOP will also describe contingency plans for continuing the study using alternate means in the event or circumstance whereby computerized systems are no longer available/viable.

III. Background/Rationale:

This SOP applies to computer systems implemented by the Data Management Center (DMC) which are used for remote data capture (electronic or other), data transmission and data storage and covers computer systems both at remote sites and at the DMC.

This SOP will address:

- System backup
- System recovery following failure or partial failure of the system
- Contingency plans for continuing the study using alternate means

IV. Procedures

<u>Backup</u>

- The Data Manager will test and verify backup procedures in accordance with testing procedures outlined in System Setup SOP - PRISM Data-008.
- The Data Manager will maintain daily backup logs (form 11.1) both at the Data Management Center and at each remote site.

Data Management Center

- The SQL Server databases in the Data Management Center will be backed up in accordance with the Data Management System Design Document.
- All other databases, documents, etc. will be automatically backed up daily at 19:30 to an external Hard Drive. ALL data on the server, including System settings will be backed up from the server.
- There will be 5 hard drives labelled Monday, Tuesday, Wednesday, Thursday, and Friday. The corresponding hard drive will be used according to the day. The Data Manager will change the hard drive every day.
- There will also be a 6th hard drive that will be used to backup the data once a month and stored off-site.

Remote Sites

- The Data Manager at the remote sites will be responsible for backing up the databases from each of the computers on a daily basis.
- Each site will have an external hard drive and each day the Data Manger will create a new folder to hold data from that day. The folder will be in the format yyyy mm dd.
- After creating the folder for the day, the data manager will back up the following databases into the folder:
    - 'PatientScheduling.mdb' from the Study Coordinator's computer
    - 'LabSpecimens.mdb' from the Lab computer
    - 'Inpatient Surveillance' database from the Inpatient Data Officer's computer
- In addition to the daily backup, the Data Manger from the Data Management Center in Kampala will remotely transfer each database to Kampala on a weekly basis. This will be done using a Smartphone in accordance with 'SOP PRISM Data-007 Using a Smartphone to transfer data from a remote site to the Kampala data center'

Recovery

- The Data Manager will investigate the cause of system failure and, if necessary, take appropriate steps to prevent similar failures .

Data Management Center

SQL Server databases

- Individual tables in the SQL Server databases are not recoverable. If a table in the database is irrevocably corrupted, the whole database would be restored.
- The most recent backup disk (from the previous day) would be retrieved. Data in the failed database would be completely deleted. The database would be restored using the restore function (from SQL) and the previous day's backup disk.
- If the backup disk from the previous day was corrupted or failed in some way, the most recent tape (from the previous day) would be retrieved. Using Backup and Restore, the backup would be transferred to disk and the database could then be re-built using restore function.

MS Access databases

- The most recent backup disk (from the previous day) would be retrieved. The database would be restored using the Windows restore program and any existing file(s) would be overwritten or deleted.
- If the backup disk from the previous day was corrupted or failed in some way, the most recent disk (from the previous day) would be retrieved. The database would be restored using the Windows restore program and any existing file(s) would be overwritten or deleted.

Remote Sites

- The most recent copy of the MS Access database on the external hard drive would be copied to the computer and any previous versions would be overwritten or deleted.

<u>**Contingency Plans**</u>

Data Management Center

- In the event that the entire computer system became permanently unavailable, data currently in the database and known to be accurate would be exported to an alternative data storage area (if necessary). If the accuracy of the data currently in the database could not be determined, the database would be restored from backup disks. The sponsor and all clinical sites would be immediately notified of the problem and the clinical sites would be informed that they could no longer import data into the system or address queries on the website until further notice.
- Depending upon the reason for the computer system being unavailable, several contingency plans could be enacted.

Some component of the system becomes irreparably unstable

- If some component of the system had become unstable and no changes, updates or patches were able to repair the problem, the component would be replaced by an alternative component (if available). For example: Some aspect of SQL failed, no fix was available and Microsoft removed SQL from the market. SQL would be removed from the system and Oracle or similar software would be substituted. The system would be re-built and re-tested (according to SOPs) prior to being released.

It is not possible to replace the component

- In the unlikely event that it is not possible to substitute some failed component in the system, that piece of the system will be removed. Where possible, all other pieces of the system will be retained. Alternative means to work around the component will be established. For example: (i). if the Microsoft Access Data Entry component of the system failed and no substitute software could be found, sites would send copies of their CRFs to a designated site to be manually entered into the system using double-keyed entry. Once the data was entered into the database it would be queried and corrected via the internet using the other pieces of the system as per the original process. (ii). If the Query Generator failed to work, forms would be entered and verified as per usual. The Query Generator would be removed from the system and data would then be manually queried using a MS Access (or similar) data query process. If possible these queries would be posted to the web-site and the rest of the data process would continue as usual.

The entire system permanently fails or becomes unviable

- Should the entire system become unstable or the sponsor refuses to continue the study using the system, two possible contingencies exist.

i. Data will be collected using traditional non-electronic data collection procedures. Sites would be asked to make copies of all CRFs and send them to the coordinating center or a designated data entry site. At San Francisco, a new database would be set up on a stable platform and all data would then be re-entered from the CRF by the manual double-key-punch method. Queries would be printed in a report format at the San Francisco and manually faxed to the sites. The site would address the queries manually on their CRF and then fax the CRF at issue back to the San Francisco where the correction would be manually entered into the data system.

ii. IDRC would relinquish data management to some other data management organization.

Remote Sites

- In the event that the computer system became permanently unavailable at a remote site (due to fire, theft, etc), the computer system would be replaced with a similar computer system.

V. Documentation

- Data Management System Design Document Version 2.0
- SOP PRISM Data-008 System Set-up
- SOP PRISM Data-007 Using a Smartphone to transfer data from a remote site to the Kampala data center

THE REPUBLIC OF UGANDA

PROGRAM FOR RESISTANCE, IMMUNOLOGY, SURVIELLANCE, AND MODELLING OF MALARIA PROJECT (PRISM)

Standard Operating Procedure

PRISM DATA-012: CHANGE CONTROL PROCEDURE

**SOP ID#: PRISM DATA-012 Version 1.0**				**DIVISION: DATA**
Written date: 22 June 2011<br>Effective date: 01 July 2011<br>SOP review cycle: Yearly<br>SOP reviewers: Data Center Director, Study Coordinators				Written by: Geoff Lavoy<br>Approved by: Edwin Charlebois
**Vers#**	**Date Modified:**	**Modified by:**	**Approved by:**	**Approved Signature:**
**Vers#**	**Date Reviewed:**	**Annual Review (no changes required) by:**		**Reviewed Signature:**

Target Audience:

Principal Investigator	X	Data Manager	X
Investigators	X	Data Officer	X
Study coordinators	X	Lab staff	X
Data Center Director	X	Local Assistants	

The following study personnel have read and understood SOP#: PRISM DATA-012:

Date	Name	Signature

THE REPUBLIC OF UGANDA

SOP ID#: PRISM DATA-012	DIVISION: DATA
Effective date: 01 July 2011	Written by: Geoff Lavoy

I. Title: Change Control Procedure

II. Purpose:

The purpose of this SOP is to outline procedures and controls designed to ensure the authenticity, integrity, and, when appropriate, the confidentiality of electronic records. It also defines the change control procedures to be followed when changing a CRF or Form. This SOP also outlines procedures and controls designed to ensure all changes are effected properly, and that there is a record of the change.

III. Background/Rationale:

All data within the PRISM study must have a date/time stamp of when the data was created. Also, any change to the data thereafter must have an audit trail to capture any change to the data. This SOP applies to all databases, CRF's or Forms used in the PRISM study. Any time a CRF or Form is changed, a Change Request Form (Form 12.1) must be filled out and submitted to the Data Management Center in Kampala.

IV. Procedures

- The Study coordinator or Investigator will be responsible for completing Form 12.1 – Change Request Form any time a CRF or Form is modified.
- Databases will not be modified unless there is a completed Change Request Form.
- The data management staff will require a minimum of 3 days notice to effect any changes.
- The Data Manager will ensure that ALL changes and /or modifications are appropriately documented.

V. Audit Trails

- Changes to the data must be accompanied by secure, computer-generated, time-stamped audit trails that independently record the time and date of any entries and/or actions that create, modify or delete records.
- Audit trails must specify who made the change, what the change was, when it was made and why it was made.
- Persons who create, modify or delete data within the computer system should not be able to modify the audit trails.
- Audit trails should be created incrementally and in chronological order. A new audit trail should not overwrite existing data.

- Where data is generated/entered by remote sites, clinical investigators should retain a certified copy of audit trails. A Certified Copy means a copy of original information that has been verified, as indicated by dated signature, as an exact copy having all of the same attributes and information as the original.
- Audit trails should be retained for the same time period as other study documentation and for the length of time the computer system is maintained. The length of time study documentation is retained and the length of time the computer system is maintained should be the same; where this is not the case, audit trails should be retained for the longer of the two periods.

VI. Version Control

- The Study Coordinator and/or Investigator will maintain strict version control of the CRF's/Forms. i.e. Any change to a CRF/Form will have a new incremental Version number.
- Version updates must be implemented at all sites simultaneously.

THE REPUBLIC OF UGANDA

UGANDA MALARIA SURVEILLANCE PROJECT – PROGRAM FOR RESISTANCE, IMMUNOLOGY, SURVIELLANCE, AND MODELLING OF MALARIA (PRISM)

Standard Operating Procedure **PRISM ENTO**

Collection of indoor mosquitoes using CDC light traps and sample processing in the PRISM study

**SOP VERSION 01:** PRISM ENTO		**DIVISION:** ENTOMOLOGY
**Effective date:** 1 APRIL2011		**Written by:** Kilama Maxwell, Steve Lindsay, and Sarah Staedke
**Date Reviewed:**	**Approved by:**	**Approved Signature:**

Training Implications:

Investigators	X
Study coordinators	X
Entomologist	X
Field assistants	X
Data staff	

The following study personnel have read and understood SOP PRISM Field–001:

Date	Name	Signature

1. **Definitions and Abbreviations**

CDC Centre for Disease Control
IRS Indoor residual spraying
LLINs Long-lasting impregnated nets

PRISM Program For Resistance, Immunity, Surveillance, and Modelling of Malaria

SOPS Standard operating procedure

WHO World Health Organization

1. **Title: Collection of indoor mosquitoes using CDC light traps in the PRISM study.**
2. **Purpose:** To describe the procedure for collecting indoor entering mosquitoes using CDC light traps.
3. **Background / Rationale:**

Classically the intensity of malaria transmission is estimated by human-landing catches, where mosquitoes are caught landing on the exposed limbs of collectors. Whilst this sampling technique is deemed acceptable by WHO (WHO 2003) we will follow WHO guidelines and ensure that ‘ *Human baits should take an appropriate and effective antimalarial prophylaxis to avoid contracting malaria during collection of biting mosquitoes. Furthermore, it is not necessary to permit mosquitoes to feed; they should be collected as soon as they settle on the skin, since it can be safely assumed that biting would normally follow.*

Goal: The primary aim of this study is to measure the intensity of malaria transmission.

Specific objectives:

- Compare the relative ability of human landing catches, CDC light trap collections and pyrethrum spray collections and exit traps combined to measure human biting rates;

Duties and Responsibilities

- Field assistants will work under the supervision of field supervisors
- Field supervisors will be responsible for all the daily field activities
- Field staff will be supervised on a day-to-day basis by Kilama Maxwell, who is under the supervision of Prof Steve Lindsay.
- Each study site will be visited once a week by the field coordinator for the 1st quota of the study
- Specimens will be transported to Kampala by field coordinator on a weekly basis for proper storage

Supplies and Materials:
Fieldwork

Hammers x 9, 3” Nails x 9kg, String x 9 bundles, Mosquito nets x 300, U2 batteries for torch lights x 9 boxes, mobile telephone in plastic bag x 13, tool belts x 9, drinking water bottles (1.5 L) x 9, map of zones x 18, clip boards x 12, wooden box for storing entomology equipment x 9, wooden box for carrying light trap batteries x 9, Cool boxes x 9, torch lights x 12, rucksack x 12, petri-dishes x 1 carton, field and lab forms x 1000, CDC light traps x 60, spare bulbs for light traps x 60, CDC light trap battery terminal sockets x 120, spare motor and fan for light traps x 20, 6V battery chargers x 30, 6V batteries for light traps x 120, rain coats x 12, umbrella x 12, pens and pencils x 9 packet, GPS equipment x 9, voltmeter x 2, four weel drive vehicle x 1, motor cycles x 12, Pooters or aspirators x 12, cotton wool x 12 rolls, black bags x 4 packs.

Laboratory
1.5ml eppendorf tube x 30 bags, lever arch files x 50, dissecting kit x 24, lab forms x 500, dissecting microscope x 6, cold light sources for microscopes x 8, compound microscope x 3, glass slides 3 cartons, cover slips 3 cartons, saline 9 ltrs, bulb pipettes 6 cartons, stapler x 6, paper puncher x 1, zip lock specimen bags x 50 packs, big size zip lock bags x30 packs, tube puncher x 18.

PROCEDURES

Field work:

1. The village local council will be notified by telephone in advance to remind householders of selected houses for catching that the study team will visit the following evening.

2. Village local council will sensitize owners of all selected houses the previous evening before the field workers visit. If the door is shut or trapping is impossible for another reason, select the nearest room with a study child, moving in a clockwise direction (from households randomly selected for the cohort studies) If there is no such house, move to the next household in a clockwise direction until a suitable house is found.

3. Select suitable rooms for the CDC light traps to be put in. This room will come from households randomly selected for the cohort studies (100 households per sub-county).These should be rooms in which a study child sleeps. Inform the house owners

the purpose of the study by reading from the information sheet and obtain documented verbal consent from the owners.

4. Determine the latitude and longitude of each sampling site using a GPS and record other household features at baseline.

5. On a catching night, hang the trap with the light one-meter above the ground at the foot end of the bed. Adjust the height so that the aluminum plate is level with your waistline (this is about a meter). Make sure that all beds or sleeping positions have a bed net;
6. Label the trap with the site code, cluster code, household number, room ID, child number, and date, by writing on a slip of paper and dropping it inside the trap cage/netting and in the side pockets if any;

7. Fix the colour-coded lead terminals from the trap to match those of the battery terminals and make sure that the fan is blowing downwards into the net and not upwards by feeling the air current from both ends (top and bottom);

8. Leave one terminal of the battery disconnected; this is to be connected by either the village local council (LC), the household head, or field worker (FW) at 19.00 hours to start operating the trap. The trap should run throughout the night;

9. In the morning at 07.00 hours, before disconnecting the battery from the trap, tie the sleeve of the net/cage first to avoid mosquitoes escaping. All traps must be collected before 10.00am;

10. Complete section 1 of the LTC Form (annex 5) on site. Record the number of people who slept in the room with the light trap;

11. Disassemble the light trap and transport it back to the lab in the wooden transport box. Batteries should be transported in a separate wooden box;

12. Put the labelled netting/cage with the mosquitoes in a cold box or black plastic bag for transportation to the lab for processing or alternatively with a sucking tube or pooter empty all the mosquitoes in the net and transfer them into labelled cups and put these cups in a cold box or shady place;

13. Transport the collections made with the cold boxes and their corresponding forms to the laboratory as soon as possible;

14. Sample delivery form (annex 6) should be filled by the field worker or field coordinator and the person in the lab should also fill to acknowledge receiving the samples;

15. Batteries used overnight should be put on charge, and the batteries should be charged for 24 hours as in battery charging log (annex 7);

16. A scheduled light trap deployment log will be followed (annex 8)

Laboratory work

1. Place nets/cages with mosquitoes directly inside a freezer at –20ºC for about 1 hour to kill all the mosquitoes;

2. Empty contents of the net/cage in a petri-dish and place the label of the trap inside before you cover it and seal it with some cellotape;

3. Sort and identify each collection morphologically using established keys [3-4] and complete section 2 of the LTC form;

4. Record the abdominal condition of all *Anopheles species* as in section 2 of LTC form (annex 5). Based on their blood meal digestion stage or abdominal condition, anophelines can be grouped as **unfed**, **freshly fed**, **half-gravid**, and **gravid** (Figure 1).

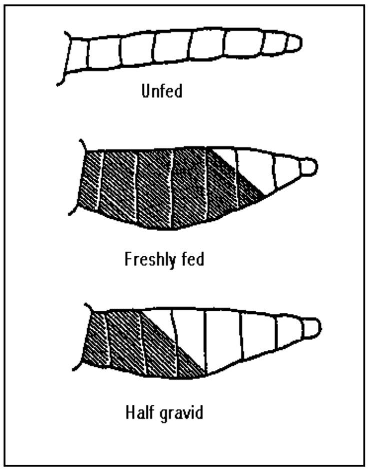

Figure 1. Abdominal conditions of a female anopheline

a) Unfed - The abdomen is flattened (this also includes sugar fed mosquitoes).
b) Freshly fed - The abdomen appears bright or dark red from the blood in the midgut. The ovaries occupy only a small area at the tip of the abdomen and this part is not red; it includes only two segments on the ventral surface and at most five segments on the dorsal surface.
c) Half-gravid - The blood is dark in colour—almost black—and occupies three to four segments on the ventral surface and six to seven on the dorsal surface of the abdomen. Ovaries occupy most of the abdomen.
d) Gravid - The blood is reduced to a small black patch on the ventral surface or may be completely digested. The ovaries occupy all the rest of the abdomen.

5. Dissecting ovaries and determining parity. Ideally 25 mosquitoes should be dissected for parity from each cluster every sampling occasion. If you find that the mosquitoes dry out too quickly making dissection impossible, the nets should be exchanged with a netting sleeve that holds a plastic beaker containing 100ml of saline. The beaker is secured in place with a rubber band.

   Equipment needed to dissect ovaries - dissecting (or stereoscopic) microscope, compound microscope, dissecting needles, fine forceps, slides, dropper and distilled water.

Parity determination is done by dissecting out the ovaries and examining them to see if they are **parous** (those that have taken a blood meal at least once and laid eggs at least once) or **nulliparous** (mosquitoes that have not taken a blood meal yet and have not laid eggs) [5].
Only females which are unfed or freshly fed are suitable for this method of parity determination. To dissect out ovaries, proceed as follows:

1. Kill the female mosquito by placing in a freezer at -20 for 1 hours Place the mosquito on a slide and add a drop of distilled water (Figure. 2).
2. While holding one needle on the thorax, pull the tip of the abdomen away from the rest of the body with another needle held in the right hand. The ovaries will come

3. Cut through the common oviduct and separate the ovaries from the rest of the specimen.
4. Transfer the ovaries to a drop of distilled water on another slide and cover with a cover slip.
5. Read the slide under a X10 magnification using a compound microscope.

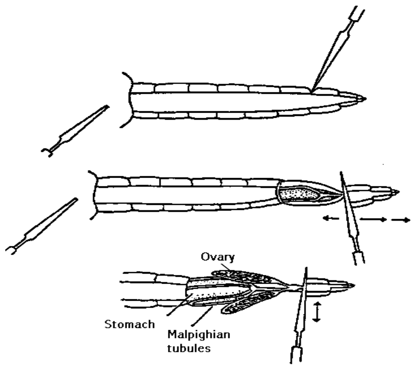

Figure 2. Dissecting ovaries

Differentiating between nulliparous and parous ovaries

- Examine the dried ovaries under a compound microscope using the 10x objective, and if necessary, confirm using the 40x objective.
- Females in which the ovaries have coiled tracheolar skeins are nulliparous (Figure 3).
- Ovaries in which the tracheoles have become stretched out are parous.
- In some females not all developed eggs are laid; if some eggs (usually less than five) are retained in the ovaries, the female is parous.

Sample storage.

All samples from the field based labs will be transported to central repository in Kampala for safe storage. A sample transmittal form (annex 9) is to be completed from the field based lab and MOLAB in Kampala.

Whole mosquito samples will be taken and stored in eppendorf tubes as detailed below for further tests. Label the tube with the study name (PRISM), site code, cluster code, household number, room ID, sample number and date of collection and stored in small

ziplock bags containing desiccant, and stored in a cupboard for future investigations. Fill in the corresponding form of the rack number, bag number, and sample numbers (annex 10).

Data entry and analysis

Entomological data will be recorded by Ugandan fieldworkers on standardised data forms. The entomologist (KM) will ensure that this information is checked in the field by the team member who collected it, or a colleague working in the same geographical area. The forms will be sent to Kampala in batches where they will also be double entered by two different data entry clerks.

Malaria Evolution in South Asia – Human Genetics Protocol #5
How to Assign IDs for the Human Genetics Community Survey

This protocol will provide the instructions for generating all IDs for households, subjects, visits and samples collected and generated during the Human Genetics community survey. These instructions must be followed exactly to make sure that the correct IDs are assigned and to avoid confusion.

Note: This protocol is only to be used with the approved human subjects protocol titled "Malaria Evolution in South Asia – Human Genetics" and not with other MESA human subjects protocols.

IDs Needed

I. Protocol ID
II. Village ID
III. Household ID
IV. Subject ID
V. Visit ID
VI. Survey Visit RDT ID
VII. Survey Visit Thin Smear ID
VIII. Survey Visit Thick Smear ID
IX. Survey Visit FTA Card ID
X. Non-Survey Visit Thin Smear ID
XI. Non-Survey Visit FTA Card ID

I. Protocol ID

1. The first two numbers of every ID (minus the DNA aliquot FreezerPro ID), is the protocol number.
2. The protocol ID for this community survey is 02.
3. The zero in the ID is important. The ID cannot start as 2.

II. Village ID

1. There are seven villages visited in this community survey. Each village has a different two digit number.
2. Here are the IDs for the seven villages:
   A. Sartheterang = 01
   B. Japijuri = 02
   C. Bachekrang = 03
   D. Jamindar = 04
   E. Rangjanphang = 05
   F. Bhaktegaon = 06
   G. Dolamora Market = 07
3. As with the protocol ID, it is very important to use both digits in the ID.

III. Household ID

1. Before starting the survey, circle all houses on a copy of the satellite map. Use a bright colored marker to circle houses.
2. Give each house a three digit number. Start in the northwest corner of the village and give the first house the number 001. Continue moving south and east to number all of the houses. Number the next house with 002.
3. Write the three digit number on the satellite map next to the house.
4. To assign the household ID at the initial study visit, find the three digit number given to each house.
5. The household ID is composed of three parts:
   A. Protocol ID (always 02 for this protocol)
   B. Village ID (see section II)
   C. Household ID
6. The order is Protocol ID**/**Village ID**/**Household ID.
7. The form of the household ID is **XX/YY/ZZZ**.
8. For example, the house numbered 075 in Bachekrang would be assigned the ID of **02/03/075**.
9. Another example is the house numbered 010 in Bhaktegaon which would have the household ID of **02/06/010**.

IV. Subject ID

1. The subject ID is the most important ID in the whole community survey. It is especially important to make sure that this ID is assigned properly.
2. Each subject enrolled will be given an individual ID. This ID will stay with them throughout the whole portion of the community survey. **The ID will not change even if they move to a different village or to a different house within the same village**.
3. The subject ID will be the household ID**/**subject number.
4. The form of the subject ID is **XX/YY/ZZZ/AA**.
5. The AA in the subject ID refers to a two digit number assigned during the initial study visit. Each member of the household will be assigned a two digit number.
6. The first household member enrolled will be assigned the number 01.
7. The second household member enrolled will be assigned the number 02.
8. People not consenting or enrolled will not be assigned subject IDs.
9. For example, the fourth person enrolled at the house with the ID 02/03/075 would be assigned the ID of **02/03/075/04**.
10. Another example is the eleventh person enrolled at the house with the ID 02/06/010 who would be given the ID of **02/06/010/11**.
11. It is important to remember that the subject ID will never change during the entire community survey.

V. Visit ID

1. The visit ID consists of three parts:
   A. Subject ID
   B. Main study visit number
   C. Day in the follow-up series

2. The form of the visit ID is **XX/YY/ZZZ/AA/B/C**.
3. The main study visit number (identified as B in point 2) refers to the number assigned to the initial study visit or repeat study visits. It only refers to visits where the Initial Visit CRF or Repeat Visit CRF is completed.
4. The initial visit will be assigned the number 1. The first repeat visit will be assigned the number 2. The next repeat visit will be assigned the number 3. Continue increasing the number as the main study visits continue.
5. The day in the follow-up series (identified as C in point 2) refers to the visits where malaria positive patients are checked to see if their parasites have cleared. The possible choices for this are:
   A. 0
   B. 2
   C. 3
   D. 5
   E. 7
6. For the main study visits where the Initial Visit or Repeat Visit CRFs are used, the day in the follow-up series will always be 0.
7. For example, the visit ID for Subject 02/06/010/11 on the initial visit will be **02/06/010/11/1/0**.
8. The visit ID for Subject 02/05/054/01 on day 3 in the follow-up series following the first repeat visit would be **02/05/054/01/2/3**.
9. The visit ID for Subject 02/05/054/01 on day 5 in the follow-up series following the third repeat study visit would be **02/05/054/01/4/5** (the third repeat study visit would be main visit number 4).
10. The visit ID for Subject 02/01/001/08 on the fourth repeat study visit with no follow-up would be **02/01/001/08/5/0** (the fourth repeat study visit would be main visit number 5).
11. No visit ID will be given for non-survey ASHA visits. Only record the Subject ID on those forms.

VI. Survey Visit RDT ID

1. The RDT ID is composed of the Visit ID and the phrase RDT.
2. The form of the RDT ID is **XX/YY/ZZZ/AA/B/C/RDT**.
3. For a RDT collected on Visit ID 02/06/010/11/1/0 the ID would be **02/06/010/11/1/0/RDT**.

VII. Survey Visit Thin Smear ID

1. The thin smear ID is composed of the Visit ID and the phrase Thin.
2. The form of the thin smear ID is **XX/YY/ZZZ/AA/B/C/Thin**.
3. For a thin smear collected on Visit ID 02/06/010/11/1/0 the ID would be **02/06/010/11/1/0/Thin**.

VIII. Survey Visit Thick Smear ID

1. The thick smear ID is composed of the Visit ID and the phrase Thick.
2. The form of the thin smear ID is **XX/YY/ZZZ/AA/B/C/Thick**.

3. For a thick smear collected on Visit ID 02/06/010/11/1/0 the ID would be **02/06/010/11/1/0/Thick**.

IX. FTA Card ID

1. The FTA card ID is composed of the Visit ID and the phrase FTA.
2. The form of the thin smear ID is **XX/YY/ZZZ/AA/B/C/FTA**.
3. For a thin smear collected on Visit ID 02/06/010/11/1/0 the ID would be **02/06/010/11/1/0/FTA**.

X. Non-Survey Visit Thin Smear ID

1. The thin smear ID from the non-survey ASHA visit is composed of the Subject ID, the phrase Thin and the date of the non-survey ASHA visit.
2. The form of the thin smear ID is **XX/YY/ZZZ/AA/Thin/DDMMYY**.
3. For a thin smear collected on 5th January, 2016 for subject 02/06/010/11, the ID would be **02/06/010/11/Thin/050116**.

XI. Non-Survey Visit FTA Card ID

1. The FTA card ID from the non-survey ASHA visit is composed of the Subject ID, the phrase FTA and the date of the non-survey ASHA visit.
2. The form of the FTA card ID is **XX/YY/ZZZ/Thin/DDMYY**.
3. For a FTA card collected on 5th January, 2016 for subject 02/06/010/11, the ID would be **02/06/010/11/FTA/050116**.

Data Access Protocol #2
Access to MESA Electronic Databases

Access to the various paper forms and paper copies of assay data was described in Data Access Protocol #1. Restricted access to the various electronic databases used by the MESA program is equally important to protect the integrity of the data in the program. This protocol will describe the various restrictions places on the three databases used in the MESA program.

REDCap Database

1. REDCap is configured to contain electronic versions of paper questionnaires and Case report forms. This database will only contain enrollment and hospital data and no experimental data.
2. REDCap has projects matching the following forms:
    A. Case report forms
    B. Inpatient treatment form
    C. Inpatient chart review form
3. Each site has its own version of each project. The projects are constructed identically between sites but access to the projects is restricted by site of employment.
4. The following staff have access to the projects corresponding to their site:
    A. Clinical research associates
    B. Site manager or site coordinator
    C. Data entry staff (if appropriate)
    D. Data entry supervisor (if appropriate)
5. The data management team will have access to projects from all sites.
6. Access to REDCap will be restricted using individual logins and passwords.
7. The lead of the data management team will be the only person authorized to create user accounts in REDCap.
8. Each member will be assigned a role in REDCap: data entry, data entry + correction, full access.
9. Data entry staff will be restricted to data entry only. They will only be able to enter data (create new records or complete unfinished records) and will not be able to edit any records already in the database.
10. Clinical research associates, the site managers and data entry supervisors will be assigned to the data entry + correction role. These staff can enter data as well as edit any record already in the database.
11. The data management team will be the only staff assigned to the full access role. This team will be allowed to enter data, edit records in the database, delete records or rename records.
12. The lead of the data management team will be the only person authorized to assign roles to various staff.

LabKey Database

1. LabKey is configured to have assays to record data determined through experiments in MESA labs.

2. It will record diverse data from diagnostics to *in vitro* culture data to genetic information.
3. Each site has its own version of each project. The projects are constructed identically between sites but access to the projects is restricted by site of employment.
4. The following staff have access to the projects corresponding to their site:
   A. Lab staff
   B. Site manager or site coordinator
   C. Data entry supervisor (if appropriate)
5. The data management team will have access to projects from all sites.
6. Access to LabKey will be restricted using individual logins and passwords.
7. The lead of the data management team will be the only person authorized to create user accounts in LabKey.
8. Each member will be assigned a role in LabKey: admin, editor, and author.
9. Lab staff will be restricted to the author role. They will be restricted to data entry. They will be able to view records that they created but not records created by other staff. They will not be able to edit data entered.
10. The site managers and data entry supervisors will be restricted to the editor role. They will be able to enter data as well as view all records created at the site they work at. They will not be able to edit data entered.
11. The data management team will be the only staff assigned to the admin role. They will be able to enter data and view all records across all of the sites. They will also be able to edit, rename and delete records.
12. The lead of the data management team will be the only person authorized to assign roles to various staff.

FreezerPro Database

1. FreezerPro is configured to have virtual freezers to match the freezers at the sites in terms of shelves, racks, boxes, etc. These virtual freezers will be used to provide an inventory of samples and time-sensitive reagents at the sites.
2. Each site will have their own freezers in the database. The freezers will be different between sites to match the exact arrangements at each site.
3. The following staff have access to the freezers corresponding to their site:
   A. Lab staff
   B. Site manager or site coordinator
   C. Data entry supervisor (if appropriate)
4. The data management team will have access to freezers from all sites.
5. Access to FreezerPro will be restricted using individualized logins and passwords.
6. The lead of the data management team will be the only person authorized to create user accounts in FreezerPro.
7. The lead of the data management team will be the only person authorized to restrict access to freezers to various site staff.
8. All staff allowed access to FreezerPro will be able to create sample records and to view and edit all sample records in freezers at their site.

9. The data management team will be the only staff allowed to move samples between sites.

Data Access Protocol #1
Storage of MESA Paper Forms and Lab Notebooks

Secure storage of informed consent forms (ICFs) and case report forms (CRFs) with restricted access based on the role in the program is essential to protecting confidential patient information. Additionally, restricted access to all paper versions of assay data is essential to protecting the integrity of collected data. This protocol will describe how the paper forms will be stored in all program offices. While the exact location may vary by site, the basic principles will be consistent between the four program sites.

Basic Document Storage

1. All program sites will have one or two offices assigned to storage of paper forms and lab notebooks. These forms include:
    A. Approved human subjects protocols and site authorization
    B. Informed consent forms
    C. Case report forms
    D. Copies of source documents, like medical charts and prescriptions
    E. Diagnostics assay forms
    F. Lab notebooks with assay data
    G. Quality control forms
    H. Lab maintenance forms
    I. Documentation related to data queries and data cleaning
2. Each office will have locks and authorized staff that regularly use the office will be the only to be issued permanent keys. Authorized staff will include:
    A. Site investigator
    B. Site manager
    C. Laboratory staff
    D. Clinical research associates (or equivalent title)
3. Other staff will be authorized to receive temporary keys when they visit the program site and require use of the office. Any keys issued will be returned by the staff when their visit is complete. The temporary staff will include:
    A. Program director
    B. Program manager
    C. Data management team members
    D. Visiting research scientists
4. Some visitors will be allowed to access the offices at the program sites but will not be issued keys. These visitors will be escorted by the site investigator, site manager or program director and include:
    A. Program sponsor or authorized representative
    B. Government of India officials or authorized representative
    C. Site investigators from other program sites
    D. Other visitors approved by sponsor or program director (approval must be issued in writing or email)
5. In the offices, paper forms will be stored in locked cabinets based on the type of form.

6. One cabinet will be dedicated to forms related to patient enrollment or medical records. Specifics of this will be described in the next section.
7. One cabinet will be dedicated to laboratory forms. Specifics of this will be described in the final section.
8. Access to the locked cabinets will be granted based on the role in the program. Specific access details for both types of cabinets will be discussed in the following sections.

Storage of Enrollment and Clinical Documents

1. All documents related to patient enrollment, questionnaires or source documents must be stored in a locked cabinet with severely restricted access.
2. These forms include all signed versions of the informed consent forms.
3. These documents will be separated into two types: those with patient names and addresses and those without.
4. Documents with patient names and addresses include informed consent forms, subject tracking forms (used for ARMD and Pathogenesis protocols) and household identification forms (used for Human Genetics protocol).
5. All of these documents will be organized by patient ID and stored in binders by increasing, sequential MESA patient ID.
6. Documents with patient names or addresses include the remainder of the case report forms (ARMD, Pathogenesis, and Human Genetics protocols), questionnaires (Pathogenesis and Human Genetics protocols) and source documentation (ARMD and Pathogenesis protocols).
7. All source documentation will have names and hospital IDs obscured and will be labeled only with the MESA patient ID.
8. Documents without names or addresses will be organized by patient ID and stored in binders by increasing, sequential MESA patient ID.
9. Permanent key issuance and access to the locked cabinets will be granted to the following staff:
    A. Site investigator
    B. Site manager
    C. Clinical research associates (or equivalent title)
10. Temporary key issuance and access to the locked cabinets will be granted to the following staff for the duration of their visit to the program site:
    A. Program director
    B. Program manager
    C. Data management team members
11. Temporary access with no key issuance (visitors will be able to request site investigator or program director to find specific documents) will be granted to the following visitors:
    A. Program sponsor or authorized representative
    B. Government of India officials or authorized representative
    C. Other visitors approved by sponsor or program director (approval must be issued in writing or email)

12. One staff member will be designated to ensure that cabinets are locked at the end of each working day. This decision will be made by the site investigator and site manager.
13. If that staff member is on vacation, the site manager will assume responsibility to ensure that the cabinets are locked at the end of each working day.

Storage of Laboratory Documents

1. All documents related to laboratory assays, lab management or quality control must be stored in a locked cabinet when lab staff leave at the end of a working day.
2. These forms include:
   A. Diagnostic assay forms
   B. Any additional assay forms developed for future assays
   C. Lab notebooks or binders from all lab staff
   D. Lab maintenance documents like freezer temperature logs, thermometer calibration logs, biosafety cabinet cleaning logs
   E. Quality control forms like RBC QC forms, media QC forms, slide reading QC forms
3. Diagnostic assay forms (ARMD, Pathogenesis and Human Genetics protocols) will be stored in binder by increasing, sequential MESA patient ID.
4. Lab notebooks and binders will be organized by staff member. The staff member can organize his/her notebooks and binders in any specific order as long as all are kept together.
5. Maintenance documents will be organized by type of form. For instance, all freezer temperature logs should be stored in the same section of a binder. Once organized by type, the forms will be ordered in chronological order.
6. Quality control forms will be organized by type of form. For instance, all media QC forms will be stored in the same section of a binder. Once organized by type, the forms will be ordered in chronological order.
7. Permanent key issuance and access to the cabinets will be granted to the following staff:
   A. Site investigator
   B. Site manager
   C. Lab staff
8. Temporary key issuance and access to the cabinets will be granted to the following staff for the duration of their visit to the program site:
   A. Program director
   B. Program manager
   C. Data management team members
   D. Approved visiting research staff that need access for training purposes
9. Temporary access with no key issuance (visitors will be able to request site investigator or program director to find specific documents) will be granted to the following visitors:
   A. Program sponsor or authorized representative
   B. Government of India officials or authorized representative

   C. Other visitors approved by sponsor or program director (approval must be issued in writing or email)

10. One staff member will be designated to ensure that cabinets are locked at the end of each working day. This decision will be made by the site investigator and site manager.
11. If that staff member is on vacation, the site manager will assume responsibility to ensure that the cabinets are locked at the end of each working day.

PNG Institute of Medical Research
Standard Operating Procedure for

Computer Backups
(ICEMR projects)

**SOP Generic Number:** 2013/V1	**Effective Date:**
**Name of Person Writing SOP:** Thomas Adiguma	**Review Date:** When necessary

**Approved by:**			
**Position**	**Name**	**Approval Date**	**Signature**
**Study Coordinator**	**Dr Leanne Robinson**		

**Previous Versions to this SOP:**		
**Version Number**	**Effective Date**	**Reason for Change**
V1		

WHAT IS THE PURPOSE OF THIS SOP?
This standard operating procedure (SOP) describes the procedure for backing up and restoring of electronic files.

WHAT STEPS MUST BE TAKEN TO COMPLY WITH THE SOP?

Steps	Responsibility	Timeframe
Select appropriate tape from rotation pool, and perform a full backup[1] of the file server[2].	System Administrator	End of every working day
Update and sign Backup Registry Log	System Administrator	After backup has been completed
Select a new tape and label as Archive Backup, and perform a full backup[1] of the file server[2].	System Administrator	At the end of every 4 week cycle
Update and sign Backup Registry Log	System Administrator	After backup has been completed
Store Archive Backup tape at off-site storage location in a fireproof cabinet	System Administrator	After backup has been completed.
Validate tapes used in rotation cycle	System Administrator	At the start of every working day after the backup was completed the previous day
Purchase and integrate new tapes into the tape rotation cycle. Record in Backup Registry Log.	System Administrator	At the start of every year
Restore file server[2] on new storage media using most recent tape backups	System Administrator	In the event of an unrecoverable failure of the file server[2]
Restore specific files to the file server[2] if requested by the developer of the files or Director	System Administrator	When requested

WHAT OTHER SOPS SHOULD BE READ AT THE SAME TIME AS THIS SOP?

TITLE OF RELATED SOPS
SOP – Filing Electronic Documents

WHAT OTHER DOCUMENTS RELATE TO THIS SOP?

Title of Related Other Documents
Backup Register Log

Guidelines for Computer Backups

PROVIDE DEFINITIONS FOR ANY NON STANDARD TERMS USED IN SOP:

1. ***Full Backup***
   The entire directory to be backed up is copied to the backup media.

2. ***File Server***
   A central computer with high capacity storage media, accessible to all networked workstations, for the secure storage of important data.

PROVIDE ANY INFORMATION WHICH MAY ASSIST PEOPLE TO CARRY OUT THE PROCEDURE

Who is Responsible
Where any of the above positions have not been appointed, or the appointed person is not available, the Principal Investigator must ensure that their responsibilities are completed by an appropriately qualified and experienced staff member.

Backup Software
The Symantec Backup Exec 2010 R2 is used at IMR for tape backup of a Windows 2008 R2 File Server.

Tape Labels
The seven rotated tapes are individually labelled *Monday, Tuesday, Wednesday, Thursday, Friday-1, Friday-2, Friday-3.* Archive tapes are labelled *Archived Backup* and the date it was created.

	Monday	Tuesday	Wednesday	Thursday	Friday
1st Week	MONDAY	TUESDAY	WEDNESDAY	THURSDAY	FRIDAY-1
2nd Week	MONDAY	TUESDAY	WEDNESDAY	THURSDAY	FRIDAY-2
3rd Week	MONDAY	TUESDAY	WEDNESDAY	THURSDAY	FRIDAY-3
4th Week	MONDAY	TUESDAY	WEDNESDAY	THURSDAY	ARCHIVED BACKUP

MONDAY to THURSDAY tapes are used on their day of the week.

FRIDAY-1 to FRIDAY-3 tapes are used on the Friday of each week.

Archived Backup tapes are used once only, then archived.

Validation of Tapes
Tapes are to be validated using facilities provided by the backup software.

Off Site Storage
Archive tapes should be stored at the Site Manager's residence, in a cool, dry, secure place and fireproof cabinet.

CSCMi

Standard Operating Procedure

**Lab Report Batch Form Workflow**	*PAGE:* 1 of 2
SOP #: **CSCMi_007** — *REVISION LEVEL:* 2	*EFFECTIVE DATE:* October 1 2012
AUTHOR(S): Steven Sullivan	*PRIMARY REVIEWER(S):* Jane Carlton

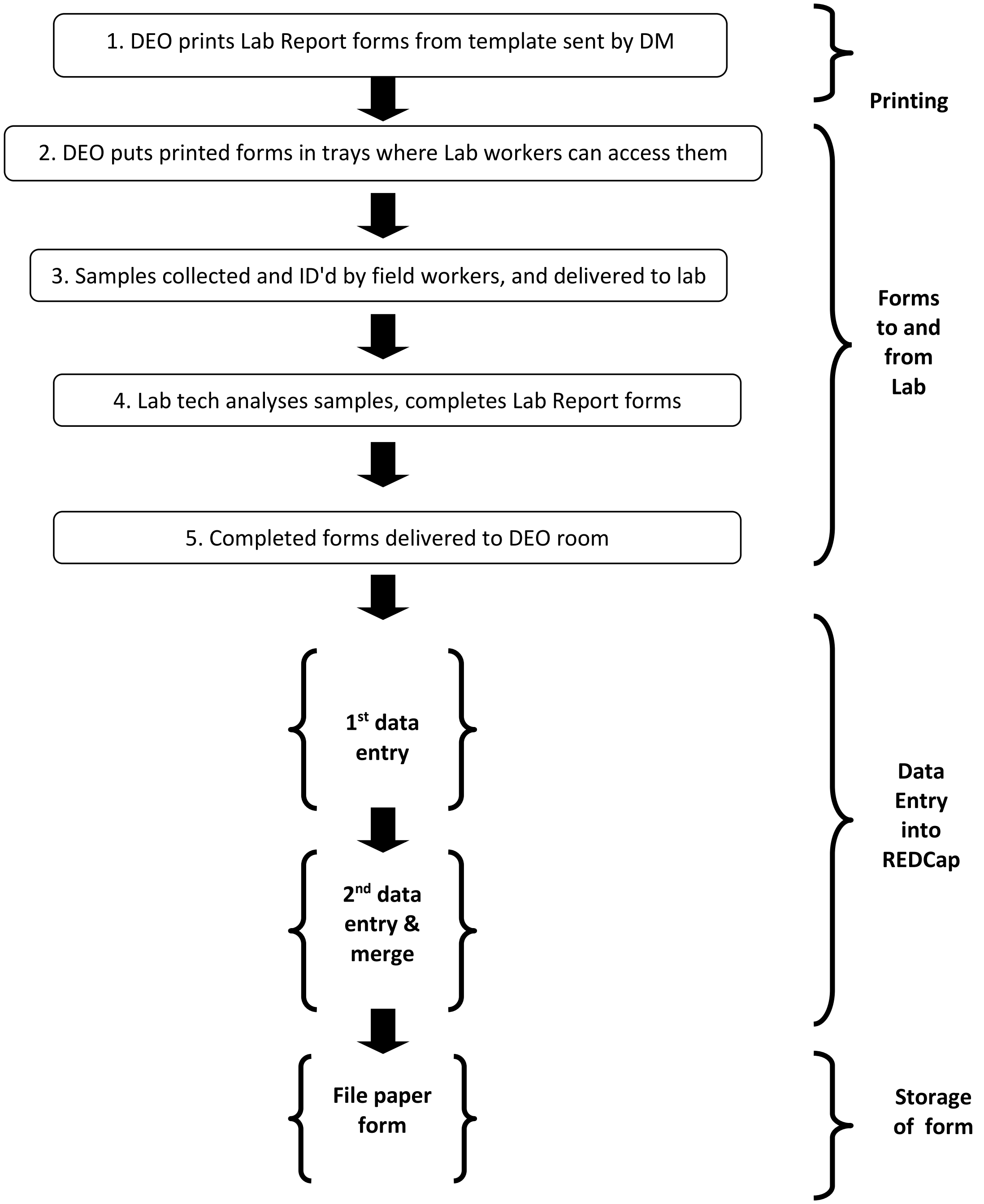

Notes on each step

(Abbreviations: DEO = Data Entry Operator; DM = Data Manager)

1. Lab Report forms are batch forms printed in 'portrait' orientation, and are single-sided. Currently there are two kinds of Lab Report forms : Microscopy and PCR.

2. Each site must set up receptacles or trays in the lab where blank forms, ready for use, can be accessed. There should be two 'in' trays, one for Microscopy forms and another for PCR forms.

3. Samples ID in the format [site code + study code + 4-digit subject ID number, *e.g*., CCX1234 for Chennai Cross Sectional study, subject# 1234] and collection date must be written on all samples by field workers. Sites must designate a person or persons to deliver the samples collected in the field, to the lab.

4. In the lab, technicians will copy the sample IDs and collection dates onto the batch form. When lab analysis is complete and all data recorded on the form, the completion date ('form date') should be written on the form and it should be placed in an 'out' tray designated for completed Lab Reports.

5. Sites must designate a person or persons who will regularly pick up the forms that are in the 'out' tray and deliver them to the DEOs for data entry.

6. Standard double data entry into REDCap is to be performed for all records, with entry one designated, *e.g*, CCX1234--1 and entry 2 designated CCX1234--2. Entry --2 should be followed immediately by DEO comparison and merging of the two entries. (REDCap will automatically designate the merged entry as CCX1234).

7. After all records on the batch form have been double entered and merged, DEOs will place it in a locked file cabinet, ordered by form type (Microscopy or PCR) and form date.

CSCMi<br>Standard Operating Procedure			
**REDCap Data Backup and Recovery**			*PAGE:* 1 of 1
SOP #: **CSCMi_017**	*REVISION LEVEL:* 1		*EFFECTIVE DATE:* October 1 2012
AUTHOR(S): Steven Sullivan		*PRIMARY REVIEWER(S):* Jane Carlton	

1. **Purpose**
   Describes the process for regular backup of REDCap data; describes process for recovery of backup data

2. **Responsibilities**
   Ensuring regular backup and (if needed) recovery of REDCap data is the responsibility of the Data Manager (DM). DM will ensure that IT personnel have set up automated backups of REDCap data and can perform recovery from them as needed.

3. **Procedure**

   I. Backup

   a. The RECap server will be set up to automatically create a gzipped (*.gz) backup copy of the database daily on a physically separate password-protected networked server. This automated process will be effected by IT personnel.
   b. Daily automated backups will be saved for one (1) month and automatically deleted after that.
   c. Every week, the DM will access the networked REDCap backup server and make a local copy of the current backup, for storage on his workstation.
   d. At the end of every month, the DM will delete all but the most recent backup from the current month.
   e. In this way, local backups from the last six (6) months will be saved. Older backups will be deleted.

   II. Recovery

   a. In the event of data loss/hardware failure, the most recent complete backup will be used to populate a new REDCap database.

CLAIM - ICEMR

DATA MANAGEMENT PLAN

CENTRO LATINO AMERICANO DE INVESTIGACIÓN EN MALARIA

(LATIN AMERICAN CENTER FOR RESEARCH IN MALARIA)

DMID PROTOCOL NUMBER:
U19AI089702

SPONSORED BY:
NATIONAL INSTITUTE OF ALLERGY AND INFECTIOUS DISEASES (NIAID)

PRINCIPAL INVESTIGATOR:
SOCRATES HERRERA, MD

PREPARED BY:
JUAN B. GUTIERREZ, PH.D.

VERSION 2.1, MARCH, 2015

ABSTRACT

This document describes the overall architecture for data management in the CLAIM ICEMR project. The objective of this document is to provide a set of standards to guide data management and analysis. This includes describing the architecture, and providing heuristics on how to use it effectively.

**System Architecture**	Juan B. Gutierrez
**High level assumptions**	The "system" is a collection of tightly integrated information systems from multiple vendors.
**Document Version**	2.1, March 2015. Updated by JBG@UGA

1 INTRODUCTION 6

1.1 PATIENT CONFIDENTIALITY 7
1.2 PERSONNEL 8
1.3 DATA ACCESS, DATA SHARING, AND DATA CONFIDENTIALITY 8
1.3.1 DATA CAPTURE 9
1.3.2 DATA REPOSITORIES 9
1.3.3 ROLES 9
1.3.4 PERMISSIONS 10
1.4 HARDWARE ARCHITECTURE 11
1.4.1 HARDWARE CONFIGURATION 11
1.4.2 SERVER SOFTWARE REQUIREMENTS 12
1.5 SOFTWARE ARCHITECTURE DIAGRAM 14
1.6 QUALITY CONTROL AND QUALITY ASSURANCE 15
1.6.1 QUALITY CONTROL 15
1.6.2 QUALITY ASSURANCE 15

2 STANDARD OPERATING PROCEDURES 17

2.1 BARCODING 17
2.1.1 PURPOSE 17
2.1.2 SCOPE 17
2.1.3 PREREQUISITES 17
2.1.4 RESPONSIBILITIES 17
2.1.5 PROCEDURE 17
2.1.6 REFERENCES 18
2.1.7 DEFINITIONS 18
2.1.8 BARCODE REFERENCE TABLE 18
2.2 QUALITY CONTROL 22
2.2.1 PURPOSE 22
2.2.2 SCOPE 22
2.2.3 PREREQUISITES 22
2.2.4 RESPONSIBILITIES 22
2.2.5 PROCEDURE 22
2.2.6 REFERENCES 22
2.2.7 DEFINITIONS 22
2.3 DATA SECURITY 24
2.3.1 PURPOSE 24
2.3.2 SCOPE 24
2.3.3 PREREQUISITES 24

2.3.4 RESPONSIBILITIES 24
2.3.5 PROCEDURE 24
2.3.6 REFERENCES 26
2.3.7 DEFINITIONS 26
2.4 WORK STATION SECURITY 27
2.4.1 PURPOSE 27
2.4.2 SCOPE 27
2.4.3 PREREQUISITES 27
2.4.4 RESPONSIBILITIES 27
2.4.5 PROCEDURE 27
2.4.6 REFERENCES 27
2.4.7 DEFINITIONS 27
2.5 FILE FOLDER NAMING 28
2.5.1 PURPOSE 28
2.5.2 SCOPE 28
2.5.3 PREREQUISITES 28
2.5.4 RESPONSIBILITIES 28
2.5.5 PROCEDURE 28
2.5.6 REFERENCES 29
2.5.7 DEFINITIONS 29
2.6 SOURCE CONTROL 30
2.6.1 PURPOSE 30
2.6.2 SCOPE 30
2.6.3 PREREQUISITES 30
2.6.4 RESPONSIBILITIES 30
2.6.5 PROCEDURE 30
2.6.6 REFERENCES 32
2.6.7 DEFINITIONS 32
2.7 WORKFLOW DOCUMENTATION 33
2.7.1 PURPOSE 33
2.7.2 SCOPE 33
2.7.3 PREREQUISITES 33
2.7.4 RESPONSIBILITIES 33
2.7.5 PROCEDURE 33
2.7.6 REFERENCES 34
2.7.7 DEFINITIONS 34
2.8 CODE COMMENTING 35
2.8.1 PURPOSE 35
2.8.2 SCOPE 35
2.8.3 PREREQUISITES 35
2.8.4 RESPONSIBILITIES 35
2.8.5 PROCEDURE 35
2.8.6 REFERENCES 36
2.8.7 DEFINITIONS 36
2.9 STORED PROCEDURE TABLE NAMING 37

2.9.1 PURPOSE 37
2.9.2 SCOPE 37
2.9.3 PREREQUISITES 37
2.9.4 RESPONSIBILITIES 37
2.9.5 PROCEDURE 37
2.9.6 REFERENCES 38
2.9.7 DEFINITIONS 38

3 STANDARDS 39

3.1 ADA COMPLIANCE CHECKLIST 39
3.1.1 ADA MOTIVATION 39
3.1.2 ADA METHODS 39
3.1.3 ADA TOOLS 40
3.1.4 ADA CHECKLIST 41
3.2 CLASS STRUCTURE 42
3.3 CUSTOM CONTROLS 45
3.4 VALIDATION 45
3.5 EXCEPTION MANAGEMENT 48
3.6 PROGRAMMING LANGUAGE STANDARDS 49
3.6.1 USE OPTION EXPLICIT 49
3.6.2 USE VARIABLE SCOPE 49
3.6.3 USE CAPITALIZATION STYLE 49
3.6.4 VARIABLE NAMING CONVENTION 51
3.6.5 CONSTANT NAMING STANDARDS 52
3.6.6 WEB CONTROL NAMING STANDARD 52
3.6.7 FUNCTIONS AND SUBROUTINES NAMING STANDARDS 53
3.6.8 ARGUMENT PASSING MECHANISM 53
3.6.9 CHOICE OF THE PASSING MECHANISM 54
3.6.10 CLASS NAMING STANDARDS 54
3.6.11 PROPERTIES NAMING STANDARDS 55
3.6.12 PARAMETER NAMING STANDARDS 55
3.6.13 ENUMERATION NAMING STANDARDS 56
3.6.14 STATIC FIELD NAMING STANDARDS 56
3.6.15 INTERFACE NAMING STANDARDS 56
3.6.16 EVENT NAMING STANDARDS 57
3.6.17 METHOD NAMING STANDARDS 57
3.6.18 SERVER-SIDE SCRIPTS 57
3.6.19 CLIENT-SIDE SCRIPTS 58
3.7 HTML STANDARDS 58
3.7.1 HTML NAMING STANDARDS 58
3.7.2 FORM ELEMENTS 58

1 Introduction

The *Centro Latinoamericano de Investigación en Malaria* (CLAIM - Latin American Center for Research in Malaria), based in Cali, Colombia, is an International Center of Excellence for Malaria Research (ICEMR) designated by NIH/NIAID to conduct malaria research non- Amazon regions of Latin America. The goal of this ICEMR is to generate knowledge and provide technical and scientific support to governments of countries members of the CLAIM to strengthen the current malaria control measures and to facilitate malaria elimination programs. CLAIMS will integrate research activities of Non-Amazonian regions in four countries: Colombia, Guatemala, Panama and Peru.

This document describes the steps taken by the Data Management (DM) and Biostatistics Core (Core B) to ensure data security, confidentiality and quality as well as provide statistical support and facilitate communication among the specific project teams and activities of all Projects and Cores (Scientific and Administrative) of CLAIM through the following specific aims:

Specific Aim 1: *Provide statistical support to country-specific ICEMR's as needed*. Core B provides support in developing protocols, and developing databases according to the needs of each project. Core B provides assistance with statistical analysis, which includes advising on approaches and methods for data analysis, running analyses with the investigators, and performing quality control checks on the analysis and use of the analysis for scientific publications.

Specific Aim 2: *Provide support for data quality efforts and data standardization*. Core B is responsible of designing and enforcing quality control procedures, full audit tracking, and data security, including hard-copy and digital information. This core also provides assistance to Administrative core in maintaining records, reports and financial accounts.

Specific Aim 3: *Provide a link to the project education and training sites*. In addition to creating meaningful opportunities for training graduate students, Core B offers training to operators and researchers in the use of technologies and in the application of best practices for data management..

Specific Aim 4: Develop and maintain a World Wide Website that serves as a research data repository and project communication center. Core B uses three WWW tools to manage data: (i) The REDCap system: it is an online application for data capture and pre-processing, (ii) DocManager: it is a centralized repository for digital information, and (iii) SharePoint: it is the system preferred to exchange files with NIAID.

1.1 Patient Confidentiality

The first consideration in designing and implementing information systems to support research involving human subjects is ethical. Research funded by the US Federal Government is subject to laws governing ethical conduct of research, under the oversight of the US Department of Health and Human Services. The conceptual framework to which the ICEMR's institutional review boards are bound is rooted in the Belmont Report (Cassell, 2000; Vollmer and Howard, 2010), according to which the driving principles of ethical research are (i) respect for persons (courtesy, respect, and transparency with patients, resulting in informed consent), (ii) beneficence (“do no harm”), and (iii) justice (reasonable, non exploitative, and fair conduct of research). This framework is regulated in the US by the Title 45, Part 46 of the Code of Federal Regulations (45 CFR 46) (Gallin and Ognibene, 2012).

However, 45 CFR 46 does not provide clear guidelines for the implementation and use of information systems. In order to have a unified framework of reference, ICEMR groups have adopted the US Health Insurance Portability and Accountability Act of 1996 (HIPAA). Title I of HIPAA is related to health insurance, and does not apply to these projects. Title II is known as the Administrative Simplification Provisions (ASP), which establishes standards for use and dissemination of health care information. Particularly, the ASP has five rules: (1) Privacy, (2) Transactions and Code Sets, (3) Security, (4) Unique Identifiers, and (5) Enforcement. In the ICEMR we adopt the Privacy and Security rules as guidelines.

The ASP Privacy Rule establishes regulations for the use and disclosure of Protected Health Information (PHI). PHI is any information concerning health status, provision of health care, or payment for health care that can be linked to an individual. PHI is defined as: (1) Names, (2) locations, (3) dates, (4) phone, (5) fax, (6) email, (7) national ID, (8) record number, (9) beneficiaries, (10) accounts, (11) certificates & licenses, (12) vehicles, (13) devices, (14) URLs, (15) IPs, (16) biometrics, (17) pictures, (18) any unique number. The ASP Privacy Rule states that research teams must: (i) Inform individuals about privacy policies, (ii) disclose information to individuals upon request, (iii) provide a mechanism for individuals to make corrections to their information, (iv) take reasonable steps to ensure confidentiality of communication, and (v) designate a privacy official and contact information.

The ASP Security Rule defines three types of security safeguards: (i) Administrative: manuals, roles, training, third parties, contingency, internal audits. (ii) Physical: hardware & software management, access, facility security plan, policies for workstation use, third party’s physical access. (iii) Technical: encryption and firewalls, audit trail, data corroboration, authentication, documentation, configuration, risk management plan.

1.2 Personnel

The personnel needed to operate the Data Management Core in the CLAIM project is comprised of 5 people as described below:

1. **Data Manager**: (1 person in the CLAIM project) This person should have and advanced quantitative degree, and should be capable of communicating effectively with researchers. This person needs to be an expert in information technology, statistics, mathematics and/or computer science.
2. **Statistician**: (1 in the CLAIM project) This person is in charge of designing and implementing reproducible analysis scripts that are feed directly from the database. This person should have an advanced degree in statistics.
3. **Programmer**: (2 in the CLAIM project) This person is in charge of designing forms in REDCap, maintaining the databases, running backups, training personnel in the use of IT tools, and providing IT support to the dta collection and analysis operation.
4. **Research Assistant**: (1 in the CLAIM project) This person is in charge of assisting the data manager and the statistician in statistical analysis and mathematical modeling. This person could be a graduate student.

1.3 Data Access, Data Sharing, and Data Confidentiality

Data originated during the course of the CLAIM project is subject to embargo until PIs have produced their publications. After the embargo period, public data release will occur according to NIH NIAID policies. Data sharing is subject to data access and data sharing policies as described in a Non-Disclosure Agreement, in which scope of the data access and purpose are clearly stated; authorship derived from the use of the data during the embargo period is also clearly defined.
In order to ensure data confidentiality, access to data is allowed via two restricted methods:

1. Through REDCap, using the built-in functionality to create users and assign permissions to view selected data fields. External users can, upon approval by the Principal Investigator, access the project database via REDCap. In general, no identifiable information is shared with external users through REDCap access.

2. Through a programmatic interface using MATLAB. Each MATLAB program must be approved by the Core B at CLAIM. These programs connect directly to the database with a designated database user that only has access to the fields required to conduct a specific type of analysis. In general, the MALAB programs approved by the Core B at CLAIM show only aggregated data. If a MATLAB program requires individual data to conduct analysis, it is compiled

and placed on the CLAIM server so that only aggregated results are shown and transmitted to external users.

1.3.1 Data Capture

Raw data is collected from multiple sources and deposited in physical and/or digital modes of support. Posteriorly, it is deposited into REDCap. Whenever raw data has physical support, a typist needs to enter this information into REDCAP via the Internet. Whenever the raw data has a digital support, a REDCap operator conducts a data import process. Data generated at the different laboratories of CLAIM will be follow the protocols and recommendations described in the Good Clinical Laboratory Practices (GCLP) guidelines defined by WHO/TDR 2008.

1.3.2 Data Repositories

Repositories are selected according to the needs of each step in the flowcharts; these repositories must meet requirements of security and confidentiality of physical and electronic data. The Data repositories are defined according to the level of access (reading, writing, erasing) that every actor has in a process. These permissions are identifiable in a matrix where each of the rows indicates an actor and in each of the columns defines a repository.
PIs and epidemiological teams of each project will have access to data sets relevant to their projects, once data cleaning and editing has finished. PIs' from each country will have access databases of their country-specific projects.
Data entry takes place on each health facility or location in the study sites of participating countries. During data entry and validation, database files are accessible by designated data entry personnel only and are password-protected.

1.3.3 Roles

The following list shows the different roles considered for the project. Note that each member of the personnel could have multiple roles, and a role could be fulfilled by multiple people. The definition of roles is necessary to facilitate definition of permissions.

- *Principal Investigator*: Responsible for monitoring the progress of the study.
- *DMP Project Manager*: Assures operation of the Web based system, coordinates operation of the members and users such that it becomes a tool for storing and sharing knowledge. Responsible for creation of CRF, protocol compliance, changes or variations. Responsible for programming (Writes programs, data capture, analysis). Responsible for managing the configuration of the database, maintain and verify the integrity of information.
- *Network Administrator*: Computer engineer. Assures functioning systems and hardware.

- *Data Quality Assurance Specialist*: Applies quality control procedures to assure data integrity
- *Biostatistics' Director*: Designs the analysis plan. Writes Scripts on every program
- *Data Entry Person*: Responsible for data entry on CRFs.
- *Study Coordinator*: Responsible for verifying integrity of the entered data.
- *Clinical Investigators*: Responsible for managing the CRFs of the study, changes in protocols, make changes on them and let them built into the system.

1.3.4 Permissions

ROLE	FUNCTIONS
**Data Manager**	Creates and manages: CRFs, Events Definitions, Rules, Sites, Subjects, Study, Extract Data Implement users and groups to Access the server's shell<br>Study Setup<br>Extract Data<br>Monitor and Manage Data<br>Submit Data
**Study Coordinator**	Create and manage: CRFs, Event Definitions, Events, Sites, Subjects, Study<br>Submit Data<br>Sing Subject Data<br>Extract Data<br>Study Setup<br>Extract Data<br>Monitor and Manage Data<br>Submit Data
**Quality Assure Coordinator**	View Datasets<br>Create Datasets<br>Cleaning Data<br>Extract Data<br>Submit Data
**Biostatistics**	View all Subjects and Events<br>Query notes and Discrepancies<br>View Datasets<br>Analysis Data<br>Extract Data
**Data Entry**	View all Subjects

**Person**	Add Subjects<br>Add new study Events<br>Enter CRF Data<br>Notes and Discrepancies<br>Submit Data
**Clinical Investigator**	View all Subjects and Events<br>View Datasets<br>Query Data

1.4 Hardware Architecture

The following section describes the minimum hardware requirements, system topology, software requirements and software architecture.

1.4.1 Hardware Configuration

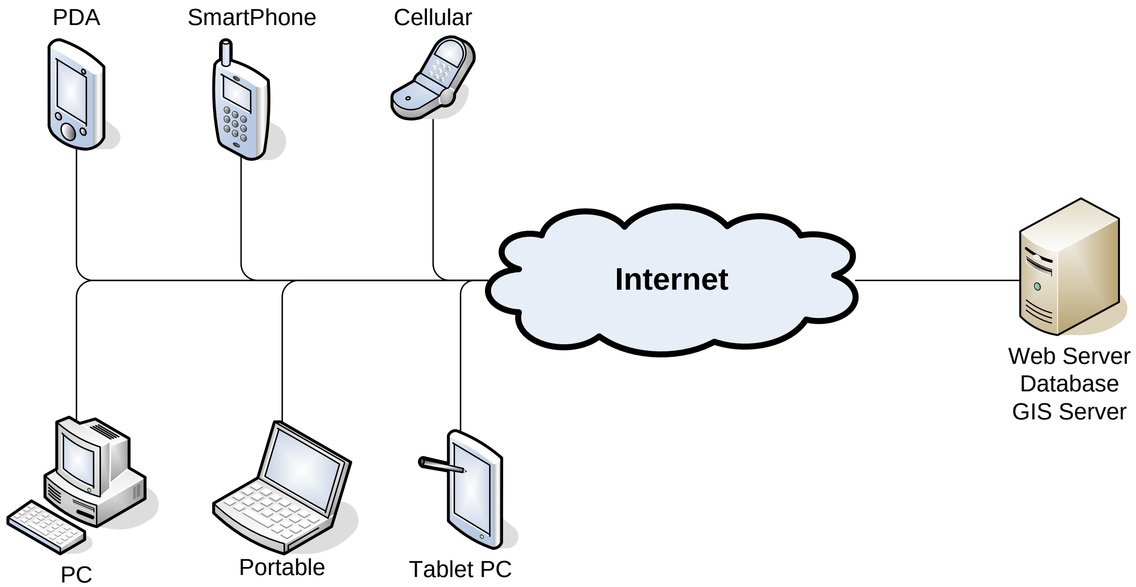

Figure 1: Different hardware devices with which the CLAIM data can be accessed.

- Dell PowerEdge Servers
- Dual Nehalem CPUs (16 Hyper threaded cores)
- Fault Tolerant RAID-10 Disk Array with Enterprise Drives

- DDR3 ECC RAM 8GB
- Gigabit uplink with a guaranteed 20mbit connection
- Fully redundant network with no single point of failure
- Multiple layers of network security
- Multiple bandwidth providers (AboveNet, ATT, Comcast, Global Crossing, Level(3), NTT)
- State of the art Data Center with backup power generators and HVAC units.
- Display

1.4.2 Server Software Requirements

- Windows Server 2012

Server I:

- o Web server with PHP (PHP 5.0.0 and up), such as Apache (any operating system) or IIS (Windows) - download PHP
- • PEAR must be installed with DB and Auth modules (how to install PEAR)
- • cURL is required for some optional components (Shared Library and Graphical Data View & Stats) - how to install cURL
- o MySQL database server (MySQL 5.0.0 and up) - how to install MySQL
- • a MySQL client (e.g. phpMyAdmin) for performing installation/upgrades
- o SMTP email server of any kind (must be configured with PHP on your web server) - often installed on web server
- o Software: REDCap 4.6.2
- o Firewall Software “Iptables”
- o Control Access List (CAL), Protected Directories, user, password.
- o Software “Open VPN”

Server II (Backup):

- DB MySQL 5.0.0
- Firewall Software “Iptables”

1.5 Software Architecture Diagram

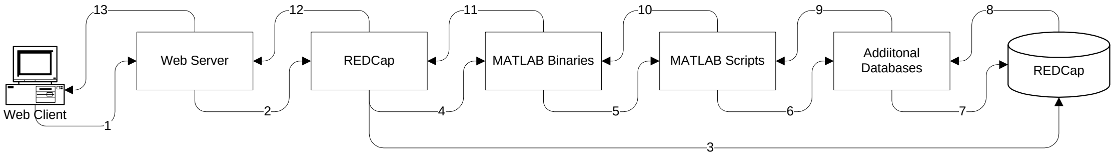

Fig. 2. Software process for analysis.

1) Web client sends request to web server.
2) Web Server starts the interface app and passes the client parameters.
3) REDcap generates data and updates the database.
4) The MATLAB DLL is initiated and passes the parameters.
5) The MATLAB scripts receive the parameters and prepares the analysis results.
6) The MATLAB scripts might consume data from additional databases.
7) Additional databases are used and are connected to the REDCap database.
8) Information for analysis is retrieved from the REDCap database.
9) Data is aggregated from multiple sources and send to MATLAB scripts for analysis.
10) MATLAB scripts process information, and, when needed, pass parameters back to the MATLAB DLL.
11) The MATLAB binaries produce an HTML output that is linked to the REDCap database.
12) REDCap sends data to the user.
13) The client program receives the information and displays it on one of the interfaces.

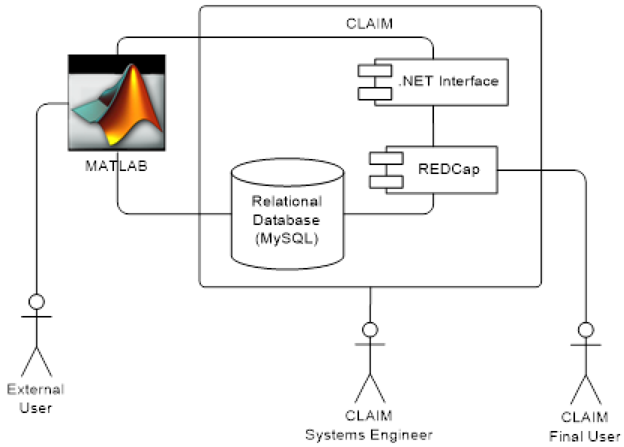

Figure 3: Topology of interaction with REDCap for analysis

1.6 Quality Control and Quality Assurance

1.6.1 Quality Control

Quality Control (QC) process consists of a series of activities that (i) provide routine and consistent checks of data completeness, integrity, and correctness, (ii) document all QA and QC activities, and (iii) assists operational procedures by identifying errors and omissions.

The SOP for QC is described later in this document.

Among the QC activities are:

1. **Training**: Operators are trained to capture and/or digitize the information correctly. Seminars are held as needed to teach operators how to use REDCap. Training reduces inconsistencies and error filling forms.
2. **QC Information System Implementation**: In the CLAIM project we use REDCap to track QC via a form designed specifically for this purpose.
3. **QC Management Plan**: The CLAIM project implements different QC procedures to ensure the quality of data according to the different stages of the project.

The QC plan is explained in the following section.

1.6.2 Quality Assurance

The Quality Assurance (QA) system is a review process conducted by personnel in roles different from the data handling roles. The QA system requires periodic tasks (often weekly) conducted throughout the duration of the process. Different QA processes are implemented according to each stage of the CLAIM project.

1.6.2.1 Data Discrepancies

The Study coordinator will be in charge of verifying every fifteen days if there are any errors or inconsistencies in the information on the REDCap platform, given the case of finding any errors, this will generate correction reports and will send them to the quality assurance coordinator of each country to make the respective data cleaning. Later the data base will be actualized and reports will be generated to be analyzed for the statistic unit.
Any problems observed in data will be reported to country-specific ICEMR team members. For this, bidirectional communication on data errors or inconsistencies, a

reporting system will be reported in master documents that will be stored at ICEMR/Core B/Master document DocManager folder. Also a system for tracking of all changes in the data records will be established. As expected volume of data is expected to be very high, double data entry will not be performed. So single data entry will be done, but our quality data assurance will be monitoring a random sub-sample of registries looking for data inconsistencies and data errors.
Data will not be disseminated until data quality has been assured. By having these extra checkpoints, the investigators of the country-specific ICEMR will able to identify causes of discrepancies in their data and address them accordingly. These steps to insure data quality are essential for data sharing, analyses, and modeling. The End User Support system at MVDC (Malaria Vaccine and Drug Development Center) will provide technical support and training for investigators in the ICEMR on the web-based REDCap system and the web-based survey system.

1.6.2.2 Handling of External Data Problems

External resources are going to be used to complement research activities and field work of different CLAIM studies. The group will obtain information of study localities through maps, data from National Malaria Control Programs (NMCP) and government reports.
These different data will be handled by the group of investigators and statisticians of the study, in the case of physical information, will be kept in locked filing cabinets in each study center; and the electronic information will have a copy stored in the DocManager program to avoid the loss of data. Discrepances of information will be documented and discussed with the institution which provided the data.

2 Standard Operating Procedures

2.1 Barcoding

2.1.1 Purpose

Barcoding of Samples. Coding of information is done using labels with barcodes with software called **Bartender v9.4**, as shown below.

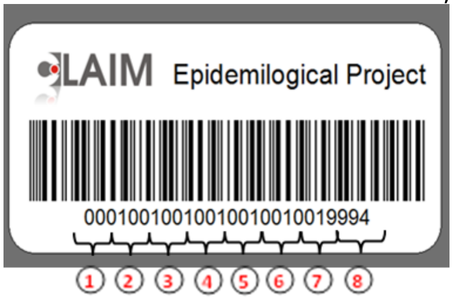

2.1.2 Scope

This SOP provides a checklist that's used to ensure the barcoding on samples is correct.

2.1.3 Prerequisites

- Bartender v9.4
- Barcoding reference table

2.1.4 Responsibilities

It is the responsibility of the personnel's taking the samples to correctly barcode the samples.

2.1.5 Procedure

1. Identify the protocol used for obtaining the sample and assign the appropriate protocol code using the Barcoding reference table.

2. Identify the project, country, study area, sentinel and survey code for the sample and assign them in the order presented in the following picture.

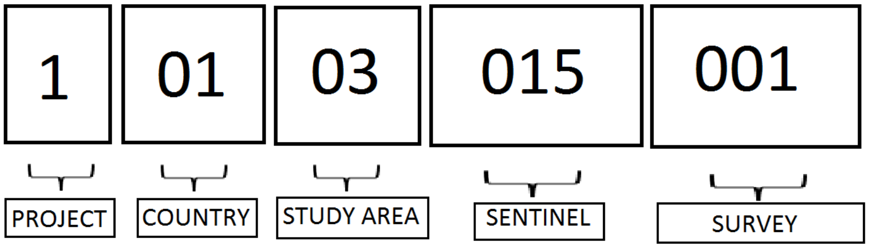

3. Obtain the ID of the sample using the 5 part bar code generated in step 2.
4. Assign the sample a 2 letter sample code and register the household # and personnel number as demonstrated in the following picture.

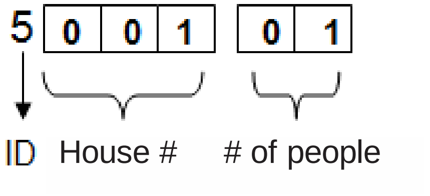

2.1.6 References

- Barcoding reference table

2.1.7 Definitions

- Coding information: The technique of providing numeric or alphanumeric codes to various processes to track and control depth of documentation and Laboratory samples and an adequate data collection.

- Numeric codes: Quantitative values assigned according to a certain number of digits to distinguish subgroups of criteria for identifying the documents pertaining to each project.

2.1.8 Barcode Reference Table

2. Protocol code: 2 decimal digits label indicating to which protocol the document type.
01: Epidemiological
02: Vectors
03: Cross-sectional study clinical profile Immune response - Pre - immune

04: Cross mosquitoes study
05: Longitudinal study
06: Relapse vivax and primaquine
07: Checks and synergism
08: Cases multilevel control
09: Control measures
10: Drug resistance mutations
11: Environmental Assessment
12: Vector Competition
13: Evaluating mosquito control measures
14: Immune response - Pre - immune
15: Mixed infections
16: severe anemia and co-infections
17: Placental Malaria

tbl_project

Project code	Name
1	Epidemiologic
2	Vectors
3	Inmunopathogenesis

tbl_country

Country code	Name
01	Colombia
02	Panama
03	Guatemala
04	Peru

tbl_study area

Study area code	Name
01	Buenaventura
02	Tierralta
03	Tumaco
04	B De Las Casas
05	Chepo
06	Bocas Del Toro
07	Piura
08	Changuinola

09	Sullana
10	Turbo

tbl_sentinel/enrollment

Sentinel code	Name
001	B. de las casas
002	A. Claras
003	Pintupo
004	Rio Diablo
005	P. Blanco
006	B. Adentro
007	Guabito
008	Tuis Tuis
009	N. Unión
010	Robles
011	P. Soldado
012	Zacarias
013	L. Delfina
014	Candelilla
015	Buchelli
016	Sullana
017	Bellavista
018	Querecotillo

tbl_survey

Survey code	Name
001	Encuesta Censo
002	Encuesta Síntomas
003	Encuesta Sociodemografica y de Capacidades, Actitudes y Prácticas
004	Descripción de Sitios de Cría de Mosquitos
005	Registro para Captura de Mosquitos con Atrayente Humano
006	Captura de Mosquitos en Sitios de Reposo
007	Captura de Mosquitos con Trampas CDC
008	Estado de la Paridad de las Hembras de Anopheles
009	Registro para Obtención de Series Entomológicas
010	Registro para Obtención de Isofamilias
011	Identificación de Material Entomológico
012	Codificación de voluntarios perfil clínico

013	Evaluación clínica de voluntarios perfil clinico

**Simple code**	**Name**
GG	Thick blood smear
PF	Filter paper
RT	Rapid diagnosis test

This section describes the software architecture, session management, object inheritance and database architecture.

2.2 Quality Control

2.2.1 Purpose

Quality control during data capture

2.2.2 Scope

This SOP provides an outline of the quality assurance procedure during data capture stage and does not cover data validation and external data problems. This SOP describes a process to detect and correct errors in a database associated to values of random variables.

2.2.3 Prerequisites

- Workstation
- Access to data depositories

2.2.4 Responsibilities

Specially assigned personnel are responsible for conducting periodic data assurance procedures.

2.2.5 Procedure

1. Let p be the total number of samples.
2. Let q be the quality assurance sample size.
3. Let n be the number of quality assurance iterations.
4. Let T be the percentage of errors in the sample universe.
5. Let $e = \frac{\tilde{q}}{q}$ be the percentage of error found during the quality assurance procedure.
6. Let Ta be the target percentage of errors in the samples.
7. Let $n = p\,(1 - T_a/e)/q$..
8. Run the quality assurance process n times.

2.2.6 References

2.2.7 Definitions

Let us define the following variables:

· p = Total number of samples.

- q = QC sample size.
- n = number of QC iterations.
- T = Percentage of erroneous data in the sample universe.
- T_a = Acceptable percentage of errors in the sample universe.

During the first iteration of QC, let $e = \frac{\tilde{q}}{q}$represent the percentage of error found during a QC procedure, where $\tilde{q}$ is the number of samples with error. The percentage of total error after finding and correcting errors in the first QC sample of size q is $T = (p - q)e/q$. After n iterations of QC, the percentage of total error is $T = (p - nq)e/q$. Let T_a represent now the target QC threshold of acceptable error. Then, the number of iterations of QC needed to bring down the error to acceptable levels is $n = p\,(1 - T_a/e)/q$. In summary, repeat QC n number of times to ensure that the whole sample is within acceptable level of statistical error.

The definition of "*acceptable error*" is context-specific. Consider the simplest case of the mean of a normally distributed random variable, $\bar{x} = \frac{1}{n}(x_1 + x_2 + \ldots + x_n)$. Assume a worse-case scenario in which there are m values having error with an upper bound of 1.5σ (i.e. data outside three standard deviations centered around the mean is filtered out). Let $\bar{x}_*$ represent the mean with error. Then, $\bar{x}_* = \frac{1}{n}(x_1 + x_2 + \ldots + x_n + \frac{3}{2}m\sigma) = \bar{x} + \frac{3m}{2n}\sigma$. Given the uncertainty associated with the measures, a hypothetical statistician determines in this example that a 1% variation in the mean is negligible. Thus, we require that $\bar{x}_* \leq 1.01\bar{x}$, then $\bar{x} + \frac{3m}{2n}\sigma \leq 1.01\bar{x}$, from where $m \leq \frac{2n}{300\,\sigma}\bar{x}$. For example, in a sample universe of 10,000 elements with a mean of 100 and a standard deviation of 50, we could accept $m \leq \frac{2 \cdot 10{,}000}{300 \cdot 50} 100 = 133.3$ records with maximum error of $1.5\sigma = 1.5 \cdot 50 = 75$ to guarantee that the mean is modified in at most 1% due to errors. That is, the acceptable number of records with error is $\frac{m}{n} = \frac{133.3}{10{,}000} = 1.3\%$.

For example, let us examine a case in which 10,000 entomological records registered in a paper-based log are entered in a computer. A QC process to test that digital data corresponds to the paper-based data would be as follows: The total number of samples is $p = 10{,}000$. Define a QC sample size of $q = 200$; this means that an operator selects randomly 200 records from the digital database and test these records against the paper-based records for correspondence. Let us say that 10 records showed inconsistencies, and use the acceptable error found in the previous paragraph. Then $T_a = 0.0133$, $e = 0.05$, and $n = p\,(1 - T_a/e)/q = 10{,}000 \cdot (1 - 0.0133/0.05)/200 = 36.7$. This means that a QC operator would have to repeat 37 times the process of selecting randomly 200 records to verify correspondence between paper and digital records, to guarantee that the calculation of the mean value of a random variable is not changed more than 1%. Note that when errors are found during QC, they are corrected. These corrected records should not be part of subsequent QC sampling, hence the condition of random selection without replacement for the QC sample mentioned above.

2.3 Data Security

2.3.1 Purpose

Secure Data Transfer via email and/or portable devices.

2.3.2 Scope

This SOP is for the general practice used to ensure data security via email and portable device.

2.3.3 Prerequisites

Knowledge of using Winrar encryption.

2.3.4 Responsibilities

All personnel are required to follow security guidelines within this SOP.

2.3.5 Procedure

- To connect to the CLAIM server, use a VPN connection.
- No patient data is to be transferred by email, if a data transfer do occur through email, the data has to be encrypted using winrar.
- To encrypt a document using WinRAR's graphic user interface on Windows:
  a. Right-click the file you want to encrypt and select the option "Add to archive..."

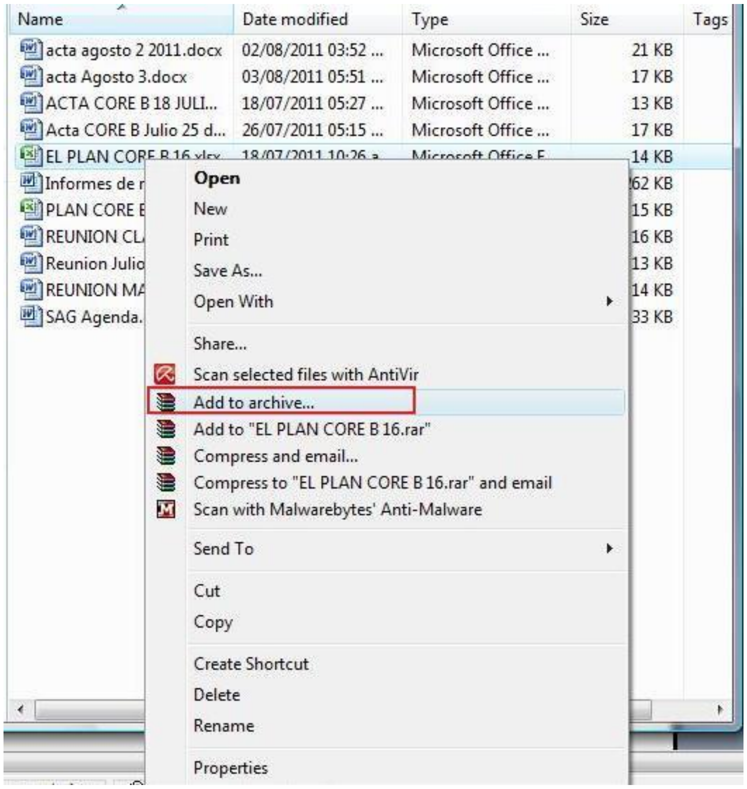

b. Select the tab "Advanced" > "Set Password". This action will display the dialog titled "Archiving with password". c. Enter the password that has been informed to you. Do not write this password anywhere, and do not transmit it

by email. d. Select "Encrypt File Names". This step is very

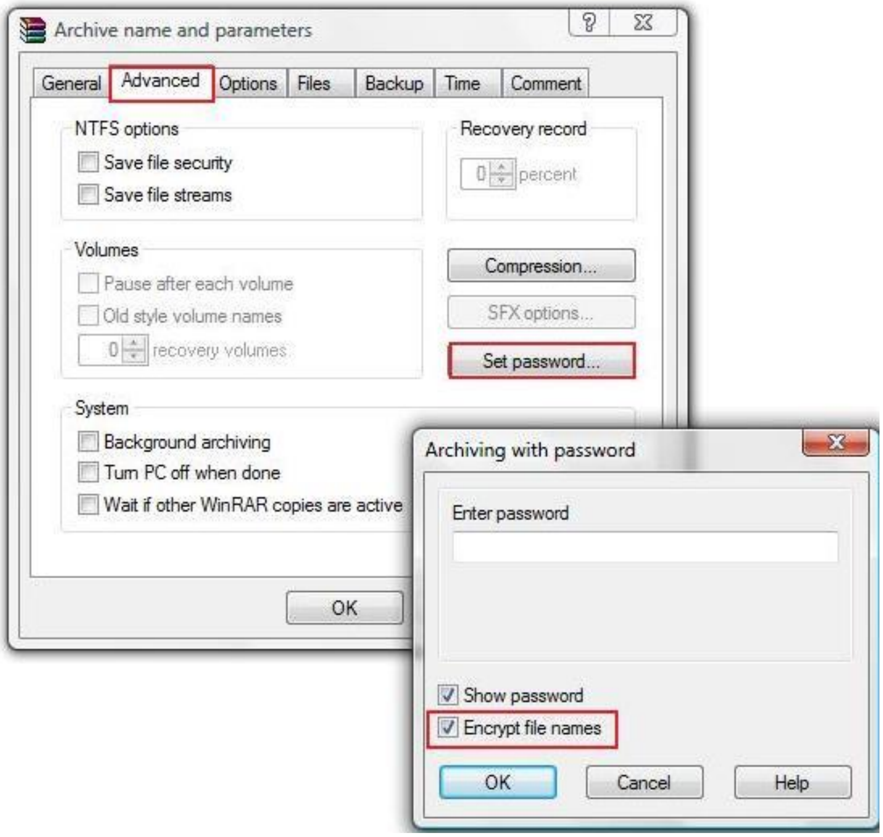

important

- When using a web browser to access email, always use http.
- When using mobile device to access email, always use SSL in the mail client.
- No patient data is to be transferred by portable device.
- Flash drives are acceptable form of data transfer ONLY for encrypted files as described above. We discourage the use of flash drives.
- Portable drives are acceptable forms of data transfer IF AND ONLY IF they provide hardware encryption. If a portable hard-drive is to be used, we strongly prefer Western Digital with WD Smartware technology (this excludes the Elements line of products). Encryption must ALWAYS be turned on.

2.3.6 References

2.3.7 Definitions

- Winrar: Data compression tool.

2.4 Work Station Security

2.4.1 Purpose

Workstation Security.

2.4.2 Scope

This SOP provides a checklist that's used to ensure Data Security using a Workstation.

2.4.3 Prerequisites

- Workstation.

2.4.4 Responsibilities

All personnel operating workstations.

2.4.5 Procedure

- Always use strong passwords, i.e. more than 10 characters combining numbers, letters and symbols and not including any dictionary word. Commit passwords to memory and never write them down. Talk by phone to receive additional instructions.
- Do not access your computer on a regular basis with administrator permissions.
- Create a user with low privileges and use it for daily operations.
- Install and run a resident antivirus. We prefer Avast. Run a boot-time scan before installing Level 3 Data software.
- Set up hard drive hardware-level encryption. This option is offered in practically all business-grade workstations. Check you workstation manual to set this up.
- Turn BitLocker on (Windows Vista and 7). If you are using a different operating system, acquire and install drive encryption technology. Your workstation must be audited via online meeting before sensitive information is shared.

2.4.6 References

2.4.7 Definitions

- Workstation: a desktop or lap top computer used for ICEMR related work.

2.5 File Folder Naming

2.5.1 Purpose

Standard folder and file naming conventions.

2.5.2 Scope

This SOP covers the standard guideline used for naming aspx files, programing convention and folder naming convention.

2.5.3 Prerequisites

1. Workstation

2.5.4 Responsibilities

The personnel who create the file or the folder are responsible for following this SOP.

2.5.5 Procedure

Naming convention for VisualBasic.NET:

1. Identify the object created as either aspx file, folder or program file.
2. If the object is an aspx file, naming it as [prefix]_[noun]_[action].aspx, where the prefix is picked from the following:

   public = All pages accessed by the public
   common = Common
   admin = Administratifve Functions
3. The action is then picked from the following:

   lst =List records
   dtl = View detail
   srch = Set search filter
   adv = View advanced search
   req = Require information to [subject]
4. The noun within the name of an aspx file would refer to the section which the file reside.
5. If the object is a program file, add prefix fun or scp to the file name depending on whether the program file is a function or a script.
6. If the object is a folder, identify the type of files that will reside within this folder. Each folder should only contain one single type of file.
7. Name the folder as a file type listed in the following:

Naming convention for MATLAB:

1. Create the following folders:
   Function = Where all function files reside
   Script = Where all scripts reside
   Data = Where intermediate data files reside
   Image = Where visualization output is stored
   IO = Where all function related to input/output (files,database,etc).
   SQL = In MATLAB, this is the folder where SQL queries are saved.
   Maps = Where SHP files are stored.
   TIFF = Where TIFF images for mapping are stored.
   Report = Where HTML reports are stored
2. Files are named with the prefix "fun" is they contain reusable code, e.g. funPrintImage, and "scp" is they contain analysis-specific steps, e.g. scpKAPLocation1_2011.

2.5.6 References

2.5.7 Definitions

- Function: a reusable piece of code that can be called from other programs.
- Script: a piece of code that performs one specific role.

2.6 Source Control

2.6.1 Purpose

Using winCVS for source control.

2.6.2 Scope

This SOP covers the procedure for obtaining winCVS and using it for source control.

2.6.3 Prerequisites

- Workstation

2.6.4 Responsibilities

The personnel modifying programs and files within in the source code depository is responsible for using this SOP for source control.

2.6.5 Procedure

1. Check if Python is installed on workstation, if not, download and install Python from http://www.python.org/.
2. Check if WinMerge is installed on workstation, if not, download and install WinMerge from http://sourceforge.net/projects/winmerge/
3. Check if WinCVS is installed on workstation, if not, download WinCVS from http://www.wincvs.org/download.html
4. Open WinCVS. Go to the menu Admin > Preferences. Select the tab title "WinCVS" and enter the following options:
    a. External Diff (checked): C:\Program Files\WinMerge\WinMergeU.exe (or the location you selected when it was installed)
    b. Default Editor: Notepad
    c. Python DLL: C:\Windows\System32\python2xx.dll (select the correct version in your system)
    d. TCL DLL: C:\Python25\DLLs\tclxx.dll (select the correct version in your system)
5. There is a dropdown in the menu; next to it to the right, there is a yellow icon of a folder. Click it and select the folder in which you want to work. The dropdown text

will show the selected folder.

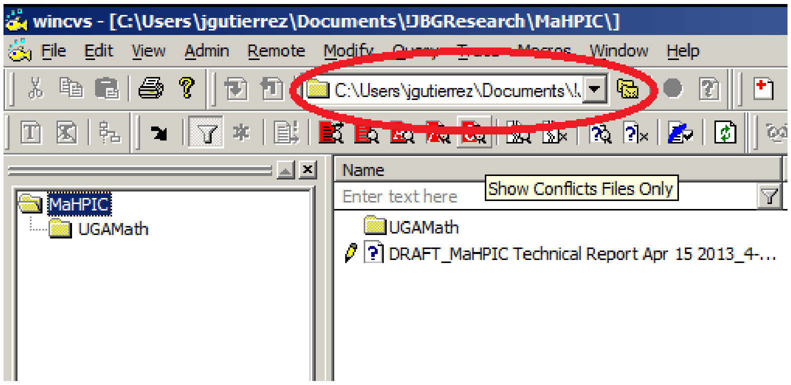

6. Go to the menu Admin > Login. In the CVSRoot field enter :pserver:[username]@euler.math.uga.edu:/BiomathRG where [username] is your assigned username. Make sure you do not have blank spaces at the end or beginning of the CVSRoot field. Click OK. A dialog window will ask your password. Enter your password and click OK.
7. On CVS command window you should see the message ***** CVS exited normally with code 0 ***** If this message is not visible, or if the code is 1, instead of code 0, there was an error. Correct it before continuing.

8. Now, you are ready to get the files. Go to the menu Remote > Checkout Module. On the field titled “Module name and path on the server” enter src. In the CVSRoot field enter :pserver:[username]@euler.math.uga.edu:/BiomathRG where [username] is your assigned username. Click OK. At this point you should see a folder with a black check-mark in the WinCVS interface.

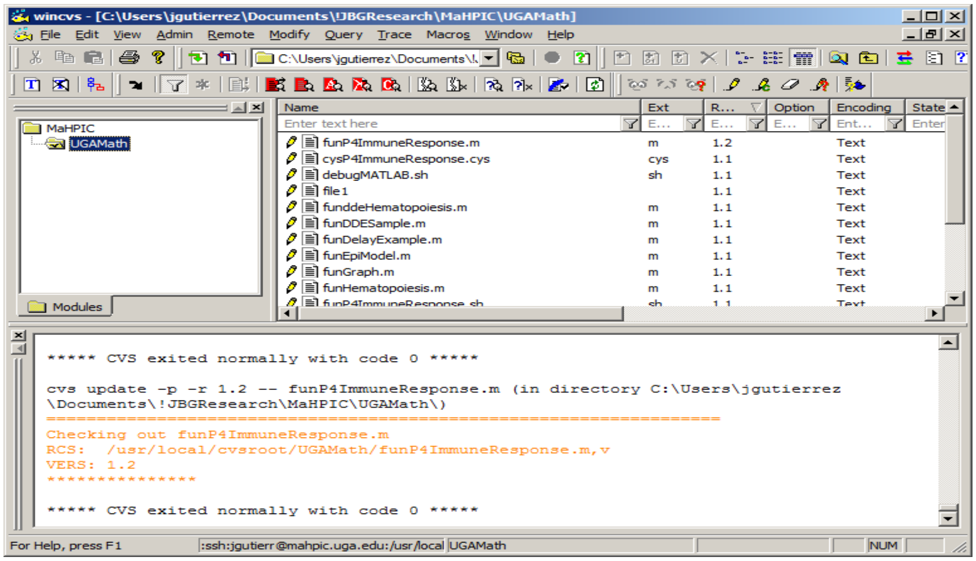

9. Once you have made changes, the file icon will change to a red color. To put your changes in the server:

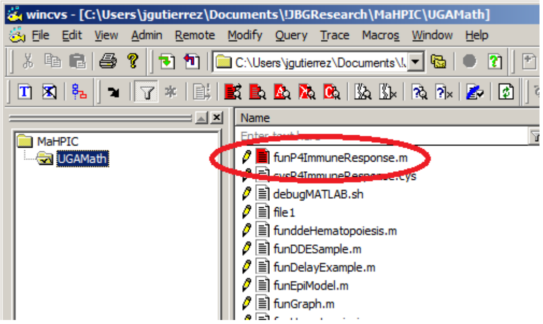

   a. Right-click and select "Update". Click OK. IMPORTANT: Always update before uploading your changes.
   b. 8.b Right-click the file and select "Commit". This will open a dialog box. IMPORTANT: Enter a comment describing your changes. It is OK to test and enter the comment "Testing CVS", but always enter a comment when committing.

10. If you want to see changes and compare versions, right-click a file and select "Graph". This will show all versions of a file. Select two versions of the same file (using CTRL+Click). You will see the differences in WinMerge.

2.6.6 References

2.6.7 Definitions

- WinMerge: Software for merging files.
- WinCVS: Software for source control.
- Python: Required language for winCVS.
- Source Control: keep multiple versions of source code

2.7 Workflow Documentation

2.7.1 Purpose

Documenting Workflow

2.7.2 Scope

This SOP is for the documentation of new workflow created. It does not cover documenting processes within the said workflow. For procedure covering documentation of processes, see the appropriate SOP.

2.7.3 Prerequisites

- List of processes covered within the workflow.
- SOP of each of the individual processes covered within the workflow. Ordered from first process to occur to last.
- List of data repositories where data will be retrieved and deposited within the workflow.
- List of actor participating within the workflow.

2.7.4 Responsibilities

The personnel responsible for documenting the workflow is responsible for obtaining the relevant prerequisites mentioned in this SOP.

2.7.5 Procedure

1. Populate the flow diagram with nodes each representing a data repository used in the workflow. Label each node with prefix DR and a number.
2. Place the node which represent the input for the first process within the workflow at the top left of the diagram and the node represent the output of the last process at the bottom right of the diagram.
3. Populate the flow diagram with directional edges representing the processes. Each edge originates from the node where the data is consumed and ends at the node where the data is deposited. Label each edge with prefix E and a number.
4. On a separate sheet, create a table with two columns. The first column contains the labels of each edge; the second column contains the SOP number corresponding to the process represented by that edge.
5. On another separate sheet, create a table with two columns, the first column contains the labels of each node, the second column contains the SOP number corresponding to the data repository represented by that node.

6. On another separate sheet, create a sheet with number of columns equal to the number of the nodes in the flow chart and number of rows equal to the number of actors. Each cell of this table, named P (for permissions), will have the permission as R (read), W (write), or RW corresponding to the actor and the data repository.

2.7.6 References

2.7.7 Definitions

- Workflow: A collection of processes.
- Processes: An action that takes data from one data repository as input, modifies the data and deposited to another data repository.
- Actors: The employee that carries out processes or workflows.

2.8 Code Commenting

2.8.1 Purpose

Standard for commenting source codes.

2.8.2 Scope

This SOP covers the guideline for commenting source codes. Software documentation exists in two forms, external and internal. External documentation, such as specifications, help files, and design documents, is maintained outside of the source code. Internal documentation is comprised of comments that developers write within the source code at development time.
Despite the availability of external documentation, source code listings should be able to stand on their own because hard-copy documentation can be misplaced. External documentation should consist of specifications, design documents, change requests, bug history, and the coding standard used.

2.8.3 Prerequisites

- Workstation

2.8.4 Responsibilities

The personnel writing the code is responsible for commenting it according to the guideline covered in this SOP.

2.8.5 Procedure

- When modifying code, always keep the commenting around it up to date.
- At the beginning of every routine, it is helpful to provide standard, boilerplate comments, indicating the routine's purpose, assumptions, and limitations. A boilerplate comment should be a brief introduction that explains why it exists and what it can do.
- Avoid adding comments at the end of a line of code; end-line comments make code more difficult to read. However, end-line comments are appropriate when annotating variable declarations, in which case, align all end-line comments at a common tab stop.
- Avoid clutter comments, such as an entire line of asterisks. Instead, use white space to separate comments from code.

- Avoid surrounding a block comment with a typographical frame. It may look attractive, but it is difficult to maintain.
- Prior to deployment, remove all temporary or extraneous comments to avoid confusion during future maintenance work.
- If you need comments to explain a complex section of code, examine the code to determine if you should rewrite it. If at all possible, do not document bad code — rewrite it. Although performance should not typically be sacrificed to make the code simpler for human consumption, a balance must be maintained between performance and maintainability.
- Use complete sentences when writing comments. Comments should clarify the code, not add ambiguity.
- Comment as you code because you will not likely have time to do it later. Also, should you get a chance to revisit code you have written, that which is obvious today probably will not be obvious six weeks from now.
- Avoid superfluous or inappropriate comments, such as humorous sidebar remarks.
- Use comments to explain the intent of the code. They should not serve as inline translations of the code.
- Comment anything that is not readily obvious in the code.
- To prevent recurring problems, always use comments on bug fixes and work-around code, especially in a team environment.
- Use comments on code that consists of loops and logic branches. These are key areas that will assist source code readers.
- Throughout the application, construct comments using a uniform style with consistent punctuation and structure.
- Separate comments from comment delimiters with white space. Doing so will make comments obvious and easy to locate when viewed without color clues.

2.8.6 References

2.8.7 Definitions

2.9 Stored Procedure Table Naming

2.9.1 Purpose

Stored procedure and table naming convention.

2.9.2 Scope

This SOP covers the guideline for naming stored procedure and tables on a relational database.

2.9.3 Prerequisites

- Workstation
- Access to SQL database

2.9.4 Responsibilities

The person creating stored procedures and tables on the database is responsible for following the guideline detailed within this SOP.

2.9.5 Procedure

1. The name of the stored procedure follows the following format: [Section]_[Table]_[Action]_[Returned Data].
2. Identify the table which the stored procedure operates on and the section the table belongs to.
3. Fill the first two part of the stored procedure name using the section and the table name obtained in step 2.
4. Identify the action that the procedure performs from the following table:

Action	Description
_Save	Insert and Update operations
_Delete	Delete operations
_Get	Retrieval data of one record
_Srch	Retrieval a list of data

Then fill in the appropriate name in the action field of the name.

5. Identify the returned data from the following table:

Action	Description
_S	Scalar

_X	XML dataset is returned
_CUR	Record set is returned

Then fill I nthe appropriate name in the returned data field of the name.

6. When naming tables, identify an name descriping the data within the table and express the name in the singular form.
7. The name of the table following the following format [prefix]_[name] where the prefix is identified from the following table:

Prefix	Data Type	Description
AM_	Numeric	Amount; represents currency
CD_	Char	A code, example; CD_AUTHORITY. All CD_ columns<br>Are maintained in the system Codes (SYSCODES) tables
DS_	Varchar	Description; represents an item in greater detail
DT_	DateTime	Date/Time; date timestamps
ID_	Long Integer	Identifier number; usually used as the primary key and foreign key
IN_	Bit	0-1 or Y-N; Boolean indicator
NM_	Varchar2	A name, example; NM_FIRST and NM_LAST
NO_	Numeric/Char	Amount others than currency, any numeric value.

2.9.6 References

2.9.7 Definitions

- Stored Procedure: Each stored procedure performs a specific function, either retrieving records or updating data on a table. Stored procedures also relay that information back to the business object.
- Table: A table within the sql database.

3 Standards

The following standards must be satisfied at the development stage. In this section it is discussed programming language standards, HTML standards, change management and source code documentation. These standards apply to IIS (web server under Windows), MATLAB, and VisualBasic.NET.

3.1 ADA Compliance Checklist

Public interfaces will comply with *Section 508 Americans with Disability Act (ADA)*. In this section, we explain the scope of this compliance, and we list the steps needed to produce a compliant web site.

3.1.1 ADA Motivation

The *Section 508 Americans with Disability Act (ADA)* gives federal civil rights protection to individuals with disabilities and guarantees equal opportunity for individuals with disabilities in public accommodations, employment, transportation, state and local government services, and telecommunications. Inaccessible technology interferes with a person's ability to locate, access, and use information quickly and easily. Section 508 seeks to eliminate barriers in information technology and to encourage development of technologies that will help achieve accessibility goals. The law specifies that agencies must give disabled employees and members of the public access to information that is comparable to the access available to others. Specifically for web developers, Section 508 requires that Federal agencies' electronic and information technology be accessible to people with disabilities, including employees and members of the public.
The criteria for web systems 508 compliance are based on access guidelines developed by the Web Accessibility Initiative of the World Wide Web Consortium. A large percentage of the requirements focus on ensuring access for people with vision impairments. These individuals often rely on assistive technologies (e.g., screen readers) that translate web-based information to audible output and refreshable Braille displays.

3.1.2 ADA Methods

The Information Technology Industry Council (ITI) is a national group committed to helping the Federal Government implement Section 508. In 2001, ITI partnered with the U.S. General Services Administration (GSA) to create a simple, Internet-based tool to assist Federal contracting and procurement officials in fulfilling the new market research requirements contained in the Section 508 implementing

regulations. These efforts resulted in the development of the Voluntary Product Accessibility Templates. Section 1194.22 of this accessibility template includes best practices for Web-based Internet Information and Applications.

- A text equivalent for every non-text element shall be provided (e.g., via "alt", "longdesc", or in element content).
- Equivalent alternatives for any multimedia presentation shall be synchronized with the presentation.
- Web pages shall be designed so that all information conveyed with color is also available without color, for example from context or markup.
- Documents shall be organized so they are readable without requiring an associated style sheet.
- Redundant text links shall be provided for each active region of a server-side image map.
- Client-side image maps shall be provided instead of server-side image maps except where the regions cannot be defined with an available geometric shape.
- Row and column headers shall be identified for data tables.
- Markup shall be used to associate data cells and header cells for data tables that have two or more logical levels of row or column headers.
- Frames shall be titled with text that facilitates frame identification and navigation
- Pages shall be designed to avoid causing the screen to flicker with a frequency greater than 2 Hz and lower than 55 Hz.
- A text-only page, with equivalent information or functionality, shall be provided to make a web site comply with the provisions of this part, when compliance cannot be accomplished in any other way. The content of the text-only page shall be updated whenever the primary page changes.
- When pages utilize scripting languages to display content, or to create interface elements, the information provided by the script shall be identified with functional text that can be read by Assistive Technology.
- When a web page requires that an applet, plug-in or other application be present on the client system to interpret page content, the page must provide a link to a plug-in or applet that complies with 1194.21(a) through (l).
- When electronic forms are designed to be completed on-line, the form shall allow people using Assistive Technology to access the information, field elements, and functionality required for completion and submission of the form, including all directions and cues.
- A method shall be provided that permits users to skip repetitive navigation links.
- When a timed response is required, the user shall be alerted and given sufficient time to indicate more time is required.

3.1.3 ADA Tools

Narrator: In order to know whether a page is accessible to a visually impaired user, try to access page’s functionality without a mouse and using Narrator. This is a Windows utility found at Programs > Accesibility > Narrator. Visually impaired

users normally use a more advanced tool (JAWS. Produced by Freedom Scientific); however, Narrator provides a good indication of accessibility.

HTML Validator: The first step to ensure ADA compliance is to comply the one of the web standards defined by the World Wide Web Consortium. We have chose HTML Transitional 4.01 because it is the most widely supported among different browsers. In order to test for compliance, use the HTML web validator at http://validator.w3.org/. Normally, it is possible to produce a web page fully compliant with the standard. If it's not, make sure that the page is accessible using assistive technology.

Bobby: Use the software Bobby to test for ADA compliance. There are 4 levels of compliance in Bobby:

- A as defined by the Web Accessibility Initiative of the World Wide Web Consortium
- AA as defined by the Web Accessibility Initiative of the World Wide Web Consortium
- AAA as defined by the Web Accessibility Initiative of the World Wide Web Consortium
- Section 508 of the Americans with Disabilities Act. When you perform the check against a page, make sure that you select the option *Section 508*.

There is a web validator at http://www.cast.org/bobby. However, it is restricted to one page every five minutes.

3.1.4 ADA Checklist

Every web page must undergo the following checks ***every time*** there is a change in the web controls. It is important to notice that small changes, apparently harmless, can easily render a page inaccessible by a visually impaired user, leading to non-compliance of the ADA requirement. In order to guarantee ADA compliance, follow the steps below:

- ✓ Check out page from source control and disable security.
- ✓ Check HTML compliance.
- ✓ Check ADA compliance.
- ✓ Access through MS Explorer the functions of the page *without* mouse *and* using Narrator
- ✓ Repeat the previous step for a Gecko-based browser, e.g. Firefox.
- ✓ Enable security and check in page.

Pay special attention to step 4 since Bobby or the W3C validator never validates JavaScript. A page could be reported as HTML and ADA compliant, and still could be inaccessible because of client-side scripts.

3.2 Class Structure

When working with classes within the system, follow the rules below:

- All relations in the database will have a corresponding class.
- The name of the entity class will approximate the name of the relation in the database with the prefix *ent*. For instance, the table *system_codes* has the associated class *entSystemCodes*.
- All entity classes will inherit from the *BaseEntityClass*.
- Create business classes to encapsulate workflows. Create one business class per each section of the system.
- Each business class will derive from the class *BaseBusinessClass*.
- Database transaction will begin, commit or roll back in the business classes.
- Per each validation rule in the system create a class that inherits from *System.Attribute*. See the section *Validation* in the *Implementation* chapter.

```
<AttributeUsage(AttributeTargets.All)> _
Public Class IsMandatory : Inherits System.Attribute
    'Atribute exists
End Class
```

For example:

The code behind is actually a class, but is considered different to entity classes and business classes because they map the user interface. These classes will follow the following directives:

1. ASP.NET Code Behind can only contain code that directly supports the display of the user interface.
2. Business logic should never be in code behind. It will be contained within in a business layer.
3. All .NET code will be written in Visual Basic.
4. Information moving from the business layer to the display code should be in the form of Objects only. If more than one item needs to be moved use a collection of objects *(ArrayList*).
5. Divide source code logically between physical files

The following class diagram depicts the class structure in the system:

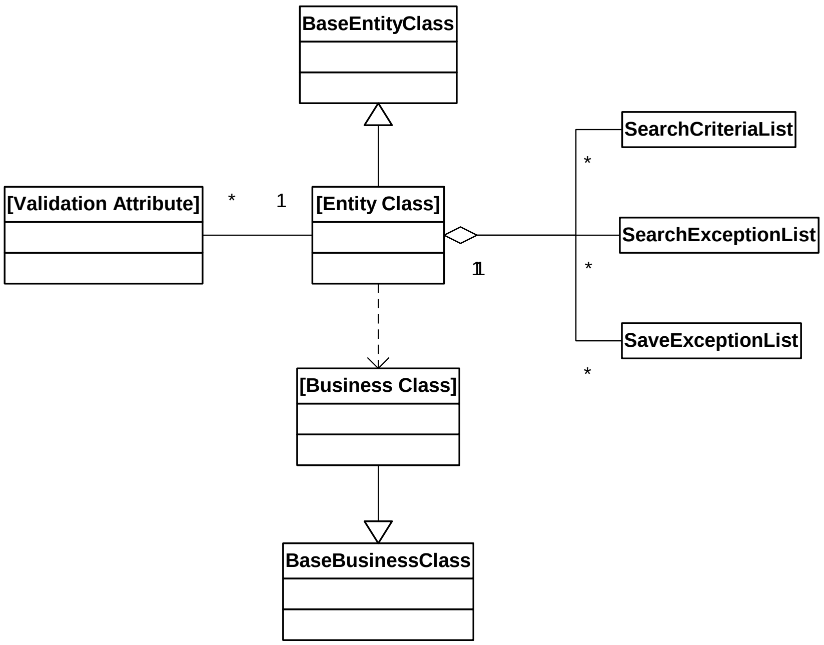


```
Search()
Delete(Transaction)
Save(Transaction)
Retrieve
```

The following methods are available on *BaseEntityClass* each class:

These methods build automatically parameterized queries based on the attributes assigned to each property in the entity class. The available attributes used to build queries are:

TableName("[table name]"): This attribute is assigned to the class. You must indicate the table name.

IsPK("[field name]"): Assign this attribute to the properties of the entity class that map primary key fields in the database.

IsDBField("[field name]"): Assign this attribute to the properties of the entity class that map primary key fields in the database

IsMandatory(): Assign this attribute to the properties whose values must be assigned before saving to the database. This corresponds to non-null fields. This attribute must be used in conjunction with *IsDBField*.

IsGeneratedID(): This attribute indicates whether the primary key for new records must be generated using the NextID stored procedure. This attribute must be used in conjunction with *IsPK*.

The following example depicts the use of attributes in an entity class:

```
<TableName("system_codes")> _
Public Class entSystemCodes : Inherits BaseEntityClass
    Private m_sType As String = ""
    Private m_sValue As String
    Private m_sDescription As String
    Private m_bActive As Boolean = True
    Private m_sTypeDescription As String
    Private m_bShowAll As Boolean = True

    <IsPK("cd_type"), ScreenLabel("System Code")> _
    Public Property Type() As String
        Get
            Return m_sType
        End Get
        Set(ByVal Value As String)
            If Value = Global.LIST_SELECT_NONE_VALUE Or Value =
Global.LIST_SELECT_ALL_VALUE Then
                m_sType = ""
            Else
                m_sType = Trim(Value)
            End If
        End Set
    End Property

    <IsPK("cd_value"), ScreenLabel("Value")> _
    Public Property Value() As String
        Get
            Return m_sValue
        End Get
        Set(ByVal Value As String)
            m_sValue = Trim(Value)
        End Set
    End Property

    <IsMandatory(), ScreenLabel("Description"), IsDBField("ds_description")>_
    Public Property Description() As String
        Get
            Return m_sDescription
        End Get
        Set(ByVal Value As String)
            m_sDescription = Trim(Value)
        End Set
    End Property
```

3.3 Custom Controls

Custom user controls are to be used for encapsulating repeated interface code. They can be added as the project grows:
Translator Control: This control uses system reflection to assign text in the correct language to every server control that has an associated entry in he table PAGE_MESSAGE.
Custom Dropdown: It is used wherever a dropdown that extract records from the database is used.
Paging Control: This control is encapsulated within the Search Results control and should never be used alone.
Search Results Control: The search results control will be used wherever there is data in list format. The search results user control has the following paging functionality:

1. Go to first page of recordset. This graphic button is visible just if the list is not displaying the first page of the recordset.
2. Go to previous page of recordset. This graphic button is visible just if the list is not displaying the first page of the recordset.
3. Go to next page of recordset. This graphic button is visible just if the list is not displaying the last page of the recordset.
4. Go to last page of recordset. This graphic button is visible just if the list is not displaying the last page of the recordset.
5. Go to a specific page within the recordset. This textbox is always visible.

Developers must create in every page where a list is needed a DataGrid with the needed layout. This DataGrid is passed by reference to the SearchResults control, along with the class that calls the search method.

```
ctlSearchResults.AssignData(grdReport, clsSyscodes)
```

3.4 Validation

Validation will be done in the server. Validation has four components:

1. Every field to be validated must correspond to a property in some entity class.
2. Every validation action will have a corresponding class that inherits from *System.Attribute*. For instance:

```
<AttributeUsage(AttributeTargets.All)> _
Public Class IsMandatory : Inherits System.Attribute
    'Atribute exists
End Class
```

3. The property must have an attribute that indicates what type of validation must be performed. For instance:

```
<IsMandatory ()> Public Property UserLogin() As String
    Get
        Return m_nUserLogin
    End Get
    Set(ByVal Value As String)
        m_nUserLogin = Value
    End Set
End Property
```

4. The validation will be done by a function that iterates though the properties using reflection.

When an exception is found, it will be added to the exception collection. There are two collections of exceptions, one for search pages, and other for detail pages.

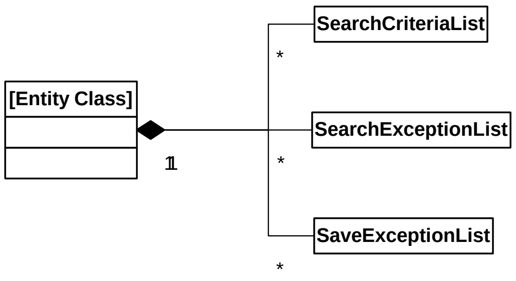

In order to show the exception on every page, place the custom control c_exception below the breadcrumbs control. For example, the search page will look something like:

```
Private Sub Page_Load(ByVal sender As System.Object, ByVal e As
System.EventArgs) Handles MyBase.Load
      If IsPostBack Then
            Dim clsSystemCodes As New system_codes
            clsSystemCodes.TypeDescription = sysType.SyscodeDescription
            clsSystemCodes.Type = sysType.SyscodeValue
            clsSystemCodes.Validate()
            cExceptionMessage.ExceptionList =
clsSystemCodes.SearchExceptionList
            Session("PAGE") = clsSystemCodes
      End If
End Sub

Private Sub lnkSearch_Click(ByVal sender As System.Object, ByVal e As
System.EventArgs) Handles lnkSearch.Click
      If cExceptionMessage.IsValid Then
Response.Redirect("dca_syscodes_lst.aspx")
End Sub
```

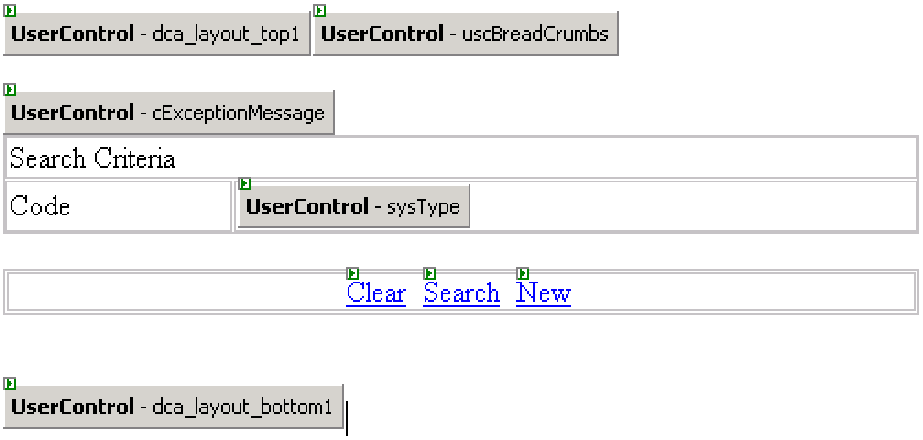

On the detail page, do the same with the custom control. The code behind will look

```
Private Sub lnkSubmit_Click(ByVal sender As System.Object, ByVal e As
System.EventArgs) Handles lnkSubmit.Click
      'Dump the values captured on screen into classes
      Dim clsSystemCodes As New system_codes
      With clsSystemCodes
            .Type = sysType.SyscodeValue
            .Value = txtValue.Text
            .Description = txtDescription.Text
            .Active = CBool(sysStatus.SyscodeValue)
            .Validate()
            cExceptionMessage.ExceptionList = .SaveExceptionList
      End With

      'If data on screen is valid, instantiate and execute business class
      If cExceptionMessage.IsValid Then
            'Instatiate bussiness class and pass entity classes as parameters
            Dim clsBizAdmin As New bizAdministration
            clsBizAdmin.SaveSystemCode(clsSystemCodes)
            'Display data error messages or success message
            cExceptionMessage.ExceptionList = clsBizAdmin.ExceptionList
      End If
End Sub
```

something like:

3.5 Exception Management

There are two types of errors: errors caused in the application and errors caused by the user. Errors caused by the user are usually associated to URL addresses being mistyped, or users trying to access restricted resources. "Access Denied" or "File not found" errors are handled with a common error page that has the same look as all other the system pages and informs the user they do not have access to the page. Add the following keys to the *web.config* file:

```
<configuration>
  <system.web>
<compilation defaultLanguage="vb" debug="true" />
<customErrors mode="On" defaultRedirect="/error/error404.aspx">
<error statusCode = "404" redirect = "/error/error404.aspx" />
</customErrors>
  </system.web>
  <appSettings>
      ...
  </appSettings>
</configuration>
```

All other exceptions must be handled by the validation controls.

3.6 Programming Language Standards

The programming language standards refer to VisualBasic.NET and MATLAB.

Naming conventions typically use a lowercase prefix or suffix to specify the variable's type and scope. The variable itself should have a meaningful name that describes what it is or what it does. Multiple-word names are concatenated, the first letter of each word is capitalized, and the underscore character is not used. If you used a variable-name template, it would be in the form *prefixNoun* or *prefixNounVerb*.

3.6.1 Use Option Explicit

The Option Explicit statement must appear in a file before any other source statements.
When Option Explicit appears in a file, you must explicitly declare all variables using the Dim, Private, Public, or ReDim statements. If you attempt to use an undeclared variable name, an error occurs at compile time.
Note: Use Option Explicit to avoid incorrectly typing the name of an existing variable or to avoid confusion in code where the scope of the variable is not clear.

3.6.2 Use Variable Scope

To determine the scope of a variable use the first letter of the variable name as the scope. A global variable starts with a lowercase `g`, a module-level variable starts with a lowercase `m`, and local variables have no prefix.

3.6.3 Use Capitalization Style

Pascal Case: The first letter in the identifier and the first letter of each subsequent concatenated word are capitalized. You can use Pascal case for identifiers of three of more characters. For Example:
Back**C**olor
Camel Case: The first letter of an identifier is lowercase and the first letter of each subsequent concatenated word is capitalized. For Example:
back**C**olor
Uppercase: All letters in the identifier are capitalized. Use this convention only for identifiers that consist of two or fewer letters.

Identifier	Case	Example
Class	Pascal	AppDomain
Enum type	Pascal	ErrorLevel

Enum values	Pascal	FatalError
Event	Pascal	ValueChange
Exception class	Pascal	WebException<br>Note Always ends with the suffix Exception.
Read-only Static field	Pascal	RedValue
Interface	Pascal	IDisposable<br>Note Always begins with the prefix I.
Method	Pascal	ToString
Namespace	Pascal	System.Drawing
Parameter	Camel	TypeName
Property	Pascal	BackColor
Protected instance field	Camel	redValue<br>Note Rarely used. A property is preferable to using a protected instance field.
Public instance field	Pascal	RedValue<br>Note Rarely used. A property is preferable to using a public instance field.

Miscellaneous

- Boolean variable names should contain Is which implies Yes/No or True/False values, such as FileIsFound.
- Avoid using terms such as Flag when naming status variables, which differ from Boolean variables in that they may have more than two possible values. Instead of DocumentFlag, use a more descriptive name such as DocumentFormatType.
- Even for a short-lived variable that may appear in only a few lines of code, still use a meaningful name. Use single-letter variable names, such as i, or j, for short-loop indexes only.
- Do not use literal numbers or literal strings, such as For i = 1 To 7. Instead, use named constants, such as For i = 1 To NUM_DAYS_IN_WEEK for ease of maintenance and understanding.
- Append computation qualifiers (Avg, Sum, Min, Max, Index) to the end of a variable name where appropriate. Use complementary pairs in variable names, such as min/max, begin/end, and open/close.
- Avoid reusing names for different elements, such as a routine called ProcessSales() and a variable called iProcessSales.
- When naming elements, avoid commonly misspelled words

- Avoid homonyms, such as write and right, when naming elements to prevent confusion during code reviews.
- Avoid elusive names that are open to subjective interpretation, such as AnalyzeThis() for a routine, or xxK8 for a variable. Such names contribute to ambiguity more than abstraction.

Examples:

Type	Name
Global string Variable	GLastName
Local Boolean Variable	BFileIsFound

3.6.4 Variable Naming Convention

Variable Type	Prefix	Example
Integer	n	nCount
String	s	sSQL
Single	f	fPayment
Long	l	lIdentification
Date	dt	dtNow
Module level	m_	m_sDescription
Array list	a	aField
Class instance	cls	clsSystemCodes

Miscellaneous

- Use prefix + Pascal case.
- Even for a short-lived variable that may appear in only a few lines of code, still use a meaningful name. Use single-letter variable names, such as i, or j, for short-loop indexes only.
- Do not use literal numbers or literal strings, such as For i = 1 To 7. Instead, use named constants, such as For i = 1 To NUM_DAYS_IN_WEEK for ease of maintenance and understanding.
- Append computation qualifiers (Avg, Sum, Min, Max, Index) to the end of a variable name where appropriate. Use complementary pairs in variable names, such as min/max, begin/end, and open/close.
- Avoid reusing names for different elements, such as a routine called ProcessSales() and a variable called nProcessSales.
- When naming elements, avoid commonly misspelled words
- Avoid homonyms, such as write and right, when naming elements to prevent confusion during code reviews.
- Avoid elusive names that are open to subjective interpretation, such as AnalyzeThis() for a routine, or xxK8 for a variable. Such names contribute to ambiguity more than abstraction.

Examples:

Type	Name
Global string Variable	gsLastName
Local Boolean Variable	bFileIsFound

3.6.5 Constant Naming Standards

Constant names should also be descriptive names in the *NOUN* or *NOUN_VERB* format. Constant names are uppercase and use an underscore character to separate words. Although there is nothing technically wrong with adding characters to constant names to specify data type and scope, it is not done often. A constant is really the same thing as a variable in the sense that both are symbolic representations of data. The difference is that variables can change and constants remain the same.
Use Capitalization Style:
Uppercase
All letters in the identifier are capitalized.
Examples:

**Type**	**Name**
Global String Constant Variable	DAY_OF_THE_WEEK
Local String Constant Variable	END_OF_WEEK

3.6.6 Web Control Naming Standard

Naming conventions typically use a lowercase prefix to specify the control. The field itself should have a meaningful name that describes what it is or what it does. Multiple-word names are concatenated, the first letter of each word is capitalized, and the underscore character is not used. If you used a form element-name template, it would be in the form *prefixNoun* or *prefixNounVerb*.

**Form Element Type**	**Prefix**
Calendar	cal
Checkbox	chk
CheckboxList	ckl
DataGrid	grd
DataList	ltd
DropdownList	sel
Form	frm
Hyperlink	lnk
Image	img
ImageButton	imb

Label	lbl
LinkButton	lnb
ListBox	lst
Panel	pnl
PlaceHolder	plc
RadioButton	opt
RadioButton	opl
Repeater	rpr
Reset	rst
Select	sel
Span	spn
Submit	sub
Table	tbl
XML	xml

3.6.7 Functions and Subroutines Naming Standards

When naming a procedure, you should use the *NounVerb* or *VerbNoun* style to create a name that clearly identifies what the procedure does. It is not necessary to use a prefix or suffix to specify the data type of the return value. Keep in mind that when you store related procedures in the same module, the Procedures box in the Code window will display those procedures alphabetically. If you stored all your data access code in a module named modDataAccessCode, you could use the *NounVerb* naming style, so related procedures are listed together. For example, the CustomerAdd, CustomerDelete, and CustomerUpdate procedures would all be displayed together in the Procedures dialog box.
Use Capitalization Style:
Pascal Case: The first letter in the identifier and the first letter of each subsequent concatenated word are capitalized. You can use Pascal case for identifiers of three of more characters. For Example:
Customer**A**dd
Uppercase: All letters in the identifier are capitalized. Use this convention only for identifiers that consist of two or fewer letters.

3.6.8 Argument Passing Mechanism

Two types of passing mechanisms:
ByRef - an argument is passed in such a way that the called procedure can change the value of a variable underlying the argument in the calling code.
ByVal - an argument is passed in such a way that the called procedure or property cannot change the value of a variable underlying the argument in the calling code

3.6.9 Choice of the Passing Mechanism

In choosing between the two passing mechanisms, the most important criterion is the exposure of calling variables to change. The advantage of passing an argument **ByRef** is that the procedure can return a value to the calling code through that argument. The advantage of passing an argument **ByVal** is that it protects a variable from being changed by the procedure.
Although the passing mechanism can also affect the performance of your code, the difference is usually insignificant. One exception to this is a value type passed **ByVal**. In this case, Visual Basic copies the entire data contents of the argument. Therefore, for a large value type such as a structure, it is more efficient to pass it **ByRef**.
Miscellaneous:

- Avoid elusive names that are open to subjective interpretation, such as AnalyzeThis() for a routine, or xxK8 for a variable. Such names contribute to ambiguity more than abstraction.
- When naming functions, include a description of the value being returned, such as GetCurrentWindowName().
- Avoid reusing names for different elements, such as a routine called ProcessSales() and a variable called iProcessSales.
- When naming elements, avoid commonly misspelled words
- Avoid homonyms, such as write and right, when naming elements to prevent confusion during code reviews.
- Specify **ByRef** and **ByVal** explicitly in VBScript function definitions.

Examples:

Type	Name
Function	GetFirstName
Subroutine	CustomerAdd

3.6.10 Class Naming Standards

The following rules outline the guidelines for naming classes:

- Use a noun or noun phrase to name a class.
- Use Pascal Case
- Use abbreviations sparingly.
- Do not use a type prefix, such as `C` for class, on a class name. For example, use the class name `FileStream` rather than `CFileStream`. Use cls as prefix for instances of classes in code.
- Do not use the underscore character (_).

- Occasionally, it is necessary to provide a class name that begins with the letter I, even though the class is not an interface. This is appropriate as long as I is the first letter of an entire word that is a part of the class name. For example, the class name `IdentityStore` is appropriate.
- Where appropriate, use a compound word to name a derived class. The second part of the derived class's name should be the name of the base class. For example, `ApplicationException` is an appropriate name for a class derived from a class named `Exception`, because `ApplicationException` is a kind of `Exception`. Use reasonable judgment in applying this rule. For example, `Button` is an appropriate name for a class derived from `Control`. Although a button is a kind of control, making `Control` a part of the class name would lengthen the name unnecessarily.

3.6.11 Properties Naming Standards

The following rules outline the naming guidelines for properties:

- Use a noun or noun phrase to name properties.
- Use Pascal Case
- Do not use Hungarian notation.
- Consider creating a property with the same name as its underlying type. For example, if you declare a property named Color, the type of the property should likewise be Color. See the example later in this topic.

3.6.12 Parameter Naming Standards

It is important to carefully follow these parameter naming guidelines because visual design tools that provide context sensitive help and class browsing functionality display method parameter names to users in the designer.

The following rules outline the naming guidelines for parameters:

- Use camel case for parameter names.
- Use descriptive parameter names. Parameter names should be descriptive enough that the name of the parameter and its type can be used to determine its meaning in most scenarios. For example, visual design tools that provide context sensitive help display method parameters to the developer as they type. The parameter names should be descriptive enough in this scenario to allow the developer to supply the correct parameters.
- Use names that describe a parameter's meaning rather than names that describe a parameter's type. Development tools should provide meaningful information about a parameter's type. Therefore, a parameter's name can be put to better use by describing meaning. Use type-based parameter names sparingly and only where it is appropriate.
- Do not use reserved parameters. Reserved parameters are private parameters that might be exposed in a future version if they are needed. Instead, if more

data is needed in a future version of your class library, add a new overload for a method.

- Do not prefix parameter names with Hungarian type notation.

Examples:

```
GetType (typeName as string ) as Type
Format (format as string, args() as object) as String
```

3.6.13 Enumeration Naming Standards

The enumeration (**Enum**) value type inherits from the Enum Class. The following rules outline the naming guidelines for enumerations:

- Use Pascal Case for **Enum** types and value names. Use abbreviations sparingly.
- Do not use an `Enum` suffix on **Enum** type names.
- Use a singular name for most **Enum** types, but use a plural name for **Enum** types that are bit fields.
- Always add the **FlagsAttribute** to a bit field **Enum** type.

3.6.14 Static Field Naming Standards

The following rules outline the naming guidelines for static fields:

- Use nouns, noun phrases, or abbreviations of nouns to name static fields.
- Use Pascal Case.
- Do not use a Hungarian notation prefix on static field names.
- It is recommended that you use static properties instead of public static fields whenever possible.

3.6.15 Interface Naming Standards

The following rules outline the naming guidelines for interfaces:

- Name interfaces with nouns or noun phrases, or adjectives that describe behavior. For example, the interface name **IComponent** uses a descriptive noun. The interface name **ICustomAttributeProvider** uses a noun phrase. The name **IPersistable** uses an adjective.
- Use Pascal Case.
- Use abbreviations sparingly.
- Prefix interface names with the letter `I`, to indicate that the type is an interface.
- Use similar names when you define a class/interface pair where the class is a standard implementation of the interface. The names should differ only by the letter `I` prefix on the interface name.
- Do not use the underscore character (_).

3.6.16 Event Naming Standards

The following rules outline the naming guidelines for events:

- Use Pascal Case.
- Do not use Hungarian notation.
- Use an `EventHandler` suffix on event handler names.
- Specify two parameters named *sender* and *e*. The *sender* parameter represents the object that raised the event. The *sender* parameter is always of type **object**, even if it is possible to use a more specific type. The state associated with the event is encapsulated in an instance of an event class named *e*. Use an appropriate and specific event class for the *e* parameter type.
- Name an event argument class with the `EventArgs` suffix.
- Consider naming events with a verb. For example, correctly named event names include **Clicked**, **Painting**, and **DroppedDown**.
- Use a gerund (the "ing" form of a verb) to create an event name that expresses the concept of pre-event, and a past-tense verb to represent post-event. For example, a **Close** event that can be canceled should have a `Closing` event and a `Closed` event. Do not use the `BeforeXxx`/`AfterXxx` naming pattern.
- Do not use a prefix or suffix on the event declaration on the type. For example, use `Close` instead of `OnClose`.
- In general, you should provide a protected method called OnXxx on types with events that can be overridden in a derived class. This method should only have the event parameter *e*, because the sender is always the instance of the type.

3.6.17 Method Naming Standards

The following rules outline the naming guidelines for methods:

- Use verbs or verb phrases to name methods.
- Use Pascal Case

Examples:

```
RemoveAll()
GetChartArray()
Invoke()
```

3.6.18 Server-side Scripts

No server side scripting is allowed. All code will reside in code behind, business classes or entity classes.

3.6.19 Client-side Scripts

All client code will be written in JavaScript. Minimize the use of JavaScript. Wherever is possible, use the Validator control. Add javascript code from page class. For instance, on the OnLoad event, the following JavaScript confirmation code can be added:

```
Private Sub Page_Load(ByVal sender As System.Object,
      ByVal e As System.EventArgs) Handles MyBase.Load
...
cmdCancel.Attributes.Add("onclick", "return confirm('Are you
      sure you want to cancel this record without saving?
      Click OK to continue.');")
...
End Sub
```

3.7 HTML Standards

The HTML standards must ensure the maximum level of accessibility without compromising needed features.

3.7.1 HTML Naming Standards

Use Capitalization Style
Pascal Case: The first letter in the identifier and the first letter of each subsequent concatenated word are capitalized. You can use Pascal case for identifiers of three of more characters. For *Example*:
  Back**C**olor
Uppercase: All letters in the identifier are capitalized. Use this convention only for identifiers that consist of two or fewer letters.

3.7.2 Form Elements

Naming conventions typically use a lowercase prefix to specify the Form Element. The field itself should have a meaningful name that describes what it is or what it does. Multiple-word names are concatenated, the first letter of each word is capitalized, and the underscore character is not used. If you used a form element-name template, it would be in the form *prefixNoun* or *prefixNounVerb*.

Form Element Type	Prefix
Checkbox	chk
Division	div
Form	frm
Frames	fra
Image Tag	img
Input Text	txt
Radio	rdo
Reset	rst
Select	sel
Span	spn
Submit	sub
Table	tbl
Table Cell (td)	td
Table Header (th)	th
Table Row (tr)	tr

Miscellaneous:
All HTML Element names and attributes should be lowercase.
`<HTML>,<HEAD>, </HEAD>, <BODY WIDTH=”650”>, </BODY>, </HTML>`
Make sure all HTML element IDs are unique and are all in lowercase.
Example:

Type	Name
Text Name Field	txtName
Radio True/False	rdoTrue, rdoFasle
Update Button	btnUpdate
